# The Multifunctional Peptide AP10W Enhances Skin Wound Healing Through Macrophage Reprogramming and Angiogenesis

**DOI:** 10.3390/biom16050720

**Published:** 2026-05-13

**Authors:** Cuiling Xuan, Zixuan Liu, Peng Zhang, Bojian Liu, Zhiqin Gao, Fei Wu

**Affiliations:** School of Life Science and Technology, Shandong Second Medical University, Weifang 261053, China; xuancuiling@stu.sdsmu.edu.cn (C.X.); liuzixuan@sdsmu.edu.cn (Z.L.); 20240064@stu.sdsmu.edu.cn (P.Z.); 20250060lbj@stu.sdsmu.edu.cn (B.L.)

**Keywords:** antimicrobial peptide AP10W, wound healing, angiogenesis, macrophage reprogramming, YAP signaling

## Abstract

Skin wound healing is a complex and highly coordinated biological process involving inflammation, cell migration and proliferation, angiogenesis, extracellular matrix remodeling and tissue regeneration. While the zebrafish-derived antimicrobial peptide AP10W exhibits broad-spectrum antimicrobial properties, its potential in tissue repair remains unexplored. Herein, we demonstrate that AP10W possesses intrinsic wound-healing capabilities, providing a preliminary investigation into its underlying mechanisms. In this study, using a full-thickness murine wound model and in vitro cell-based assays to evaluate the effects of AP10W on fibroblasts, keratinocytes, endothelial cells, and macrophages, we found that AP10W significantly promoted fibroblast and keratinocyte migration and proliferation. Furthermore, it enhanced endothelial cell motility, survival, and tube formation, while upregulating key pro-angiogenic factors, including *Vascular endothelial growth factor A* (*VEGFA*), *Platelet-derived growth factor* (*PDGF*), and *Fibroblast growth factor 2* (*FGF2*). Concurrently, AP10W drove macrophage reprogramming from a pro-inflammatory M1 phenotype toward a pro-healing M2 state, as evidenced by upregulated *Arginase-1* (*Arg-1*) and *Interleukin-10* (*Il-10*) expression, alongside attenuated *Tumor necrosis factor-alpha* (*Tnf-α*), *Interleukin-1 beta* (*Il-1β*), *Interleukin-6* (*Il-6*), and *Inducible nitric oxide synthase* (*iNOS*) levels. In vivo, the topical application of AP10W accelerated wound closure, markedly improving re-epithelialization, collagen deposition, vascularization, tissue perfusion, and skin appendage regeneration. Preliminary mechanistic studies revealed that AP10W increased YAP expression and nuclear translocation; conversely, the pharmacological inhibition of YAP significantly abrogated these pro-healing effects. Collectively, our findings identify AP10W as a multifunctional peptide with potent wound-healing properties, positioning it as a promising candidate for wound therapy.

## 1. Introduction

As the body’s main external barrier, the skin is a highly organized structure composed of a keratinized epidermis and a collagen-rich dermis [1]. It serves as a primary physical barrier against environmental insults such as mechanical damage, extreme temperature, microbial infection, and ultraviolet radiation [1]. Beyond its barrier function, the skin acts as a dynamic immune–mechanical interface that coordinates a range of homeostatic and innate adaptive immune responses to prevent pathogen invasion and maintain systemic integrity [1]. Upon disruption of the skin’s protective barrier, injuries induced by this damage induced by mechanical injury, microbial invasion/infection, chemical or thermal stimuli, and inflammation or oxidative stress can penetrate not only the epidermis but also the dermis and even subcutaneous tissues, thereby challenging the wound healing process [2,3,4]. Under physiological conditions, wound healing is a dynamic process consisting of four overlapping stages: hemostasis, inflammation, proliferation, and tissue remodeling. These phases are regulated by different but interconnected cellular and molecular events. In particular, the inflammatory phase is dominated by the immune response, whereas fibroblast proliferation, re-epithelialization, and angiogenesis are more prominent during the proliferative phase [5,6,7]. Successful wound repair therefore requires the coordinated participation of multiple cell types, including keratinocytes, fibroblasts, endothelial cells, and macrophages, which collectively contribute to epithelial restoration, granulation tissue formation, vascular reconstruction, and resolution of inflammation [1,4]. Accordingly, therapeutic strategies that simultaneously control microbial invasion, modulate inflammation, and promote tissue regeneration are highly desirable for effective wound repair [2]. In this context, antimicrobial peptides (AMPs), which possess both antimicrobial and immunoregulatory properties, have attracted increasing attention as multifunctional candidates for wound healing [8].

Antimicrobial peptides (AMPs), typically 10 to 50 amino acids in length, are a class of small, naturally occurring peptides widely present in eukaryotes, bacteria and archaea and have been recognized as promising therapeutic candidates owing to their multifunctional roles [8]. They exhibit broad-spectrum antimicrobial effects directly against bacteria, viruses, fungi and protozoa, through diverse mechanisms such as membrane disruption, inhibition of cell wall and nucleic acid synthesis, induction of oxidative stress, and interference with cell division [9,10,11,12]. Crucially, AMPs also exert potent immunomodulatory and pro-healing effects. They modulate key inflammatory cytokines and chemokines, such as IL-1β, IL-6, TNF-α and IL-10, and promote a shift in macrophage polarization from a pro-inflammatory M1 phenotype toward the pro-healing M2 phenotype, thereby facilitating inflammation resolution and tissue repair [13,14]. Furthermore, several AMPs, including AMP-IBP5, Tylotoin, CW49, Temporin and Pt5-1c, directly enhance tissue repair by stimulating the migration and proliferation of keratinocytes and fibroblasts, thereby enhancing re-epithelialization, increasing collagen deposition, and regulating matrix metalloproteinase (MMP) activity [14,15,16,17]. Occasionally, their support of angiogenesis, through the upregulation of pro-angiogenic factors like FGF, PDGF, and VEGF, ensures adequate nutrients and oxygen supply to the healing tissue [18].

The Hippo pathway is an evolutionarily conserved signaling pathway with key roles in organ development, epithelial homeostasis, tissue regeneration, wound healing and immune modulation [19,20]. Yes-associated protein (YAP), as a core downstream transcriptional co-activator in this pathway, regulates cell proliferation, survival, and migration, playing a decisive role in organ size control and regeneration [21,22]. Recent work has demonstrated that YAP activation is essential for wound healing, as it is strongly expressed in the nuclei of epidermal and dermal cells after injury and its loss dramatically impairs wound closure [19]. For instance, the small molecule YAP activator PY-60 accelerates regenerative wound repair in pig and human models by enacting a reversible pro-proliferative transcriptional program in skin cells [22]. Similarly, human adipose tissue-derived exosomes (Exo-ATs) enhance skin wound healing by targeting the Hippo-YAP axis, promoting the migration and proliferation of keratinocytes and fibroblasts, as well as collagen production [23]. Furthermore, YAP acts as an essential co-transcription factor in endothelial cells that transduces VEGF signaling into specific transcriptional programs necessary for angiogenesis. However, the potential intersection between the pro-healing functions of AMPs and the pivotal YAP pathway, specifically whether AMPs mediate repair via Hippo-YAP modulation, remains largely unknown.

Our previous studies identified AP10W, a 10-amino-acid peptide derived from the zebrafish AP-2 complex subunit mu-A (AP2M1A), which exhibits broad-spectrum antimicrobial activity against both bacteria and fungi with minimal toxicity to mammalian cells [24,25]. AP10W functions via a combined mode of action, including interaction with the microbial cell wall components, membrane depolarization, and induction of intracellular ROS [24,25]. Very recently, we demonstrated that AP10W accelerates the healing of *Candida albicans*-infected wounds, an effect that was primarily attributed to its potent fungicidal activity [24]. However, whether AP10W also exerts intrinsic pro-healing effects independent of its antimicrobial action remains unknown. This distinction is important, because improved healing in an infected wound may result not only from pathogen elimination but also from direct regulation of cellular processes involved in tissue repair. Therefore, the aim of the present study was to systematically investigate the intrinsic pro-healing potential and underlying mechanisms using four types of skin repair cells, including keratinocytes, fibroblasts, endothelial cells, and macrophages, as well as a murine full-thickness wound model in vitro and in vivo, respectively.

## 2. Materials and Methods

### 2.1. Antimicrobial Peptide AP10W

The antimicrobial peptide AP10W, with the amino acid sequence WKIKRWAIWK (observed molecular weight: 1414 Da), was synthesized by Sangon Biotech Co., Ltd. (Shanghai, China), using standard solid-phase 9-fluorenylmethoxy carbonyl (Fmoc) chemistry, as previously described [24]. The synthesized AP10W was purified >95% purity by reverse-phase high-performance liquid chromatography (HPLC) and verified using a mass spectrometer. Final products were lyophilized and stored at −80 °C until use.

### 2.2. Cell Culture

Mouse fibroblast (L929) cells and mouse macrophage (Raw 264.7) cells were cultured in high-glucose DMEM medium supplemented with 10% (*v*/*v*) fetal bovine serum (FBS) (Gibco, Grand Island, NY, USA) and 1% penicillin–streptomycin solution (P/S) (Cytiva, Marlborough, MA, USA) [26]. Both human epidermal keratinocytes (HaCaT) and human umbilical vein endothelial cells (HUVECs) were cultured according to previously established protocols [18,27]. All cell lines were incubated at 37 °C in a humidified atmosphere containing 5% CO_2_, with above 95% humidity. The culture medium was replaced every other day, and cells were passaged upon reaching approximately 80% confluence.

### 2.3. Scratch Wound Healing Assay

Cell migration was assessed using a scratch wound healing assay [28]. Briefly, L929 and HaCaT cells were seeded into 6-well plates at a density of 5 × 10^5^ cells per well and cultured to full confluence at 37 °C. Following serum starvation for 12 h in FBS-free medium, a uniform scratch was created in the cell monolayer using a 200 μL sterile pipette tip. The dislodged cells were removed by washing with phosphate-buffered saline (PBS). Subsequently, fresh medium containing 0.5% FBS, with or without AP10W, was added. Wound closure was monitored, and images were captured using an optical microscope (Nikon Eclipse Ts2, Tokyo, Japan) at 0, 24, and 48 h, respectively. Wound closure was assessed by measuring the scratch area at different time points with ImageJ software (Version 1.52, National Institutes of Health, Bethesda, MD, USA). For each image, the scale was calibrated according to the embedded micrometer. The wound boundary was manually traced using the Polygon selection tool, and the area was measured. Repair rate of scratch (%) = (C_0_ − C_t_)/C_0_ × 100%, where C_0_ represents the scratch area at 0 h and C_t_ represents the scratch area at specific times.

### 2.4. Transwell Assay

Directed cell migration (chemotaxis) was quantified using a transwell assay. Briefly, both L929 and HaCaT cells, as well as HUVECs, were resuspended in the medium containing 2% FBS supplemented with various concentrations of AP10W (0, 0.5, 0.625, 0.75, 1, 1.25 μg/mL). The cell suspension was then seeded into the upper chamber of a transwell cell culture insert at a density of 3 × 10^4^ cells per well. A total of 600 μL medium containing 20% FBS was added to the lower chamber of the transwell cell culture insert. After incubation for 24 h, cells that had migrated through the membrane were fixed with 4% paraformaldehyde (PFA) (Solarbio, Beijing, China) for 20 min, followed by staining with 0.1% crystal violet (Solarbio, Beijing, China) for another 20 min. Cell images were captured using an optical microscope (Olympus BX53E2C, Tokyo, Japan), and the number of migrated cells was quantified using ImageJ software (NIH, Bethesda, MD, USA).

### 2.5. Cell Proliferation Assay

Both 5-ethynyl-2′-deoxyuridine (EdU) incorporation and 3-[4,5-dimethyl-2-thiazolyl]-2,5-diphenyl-2-H-tetrazolium bromide (MTT) assays were performed to assess the proliferative capacity of HUVECs, L929 and HaCaT cells. For the EdU incorporation assay, immunocytochemical detection of EdU-incorporated cellular DNA was conducted using the Yefluor 594 EdU Imaging kit (Yeasen, Shanghai, China) in accordance with the manufacturer’s instructions [18]. Briefly, L929 cells, HaCaT cells, and HUVECs were seeded into 24-well plates at a density of 5 × 10^4^ cells per well. After cell attachment, different concentrations of AP10W (0, 0.5, 0.625, 0.75, 1, and 1.25 μg/mL) were added for 24 h. The cells were then exposed to 10 μM EdU for 2 h to label proliferating cells. Cells were fixed with 4% PFA for 30 min, followed by neutralization with 2 mg/mL glycine solution for 5 min. The fixed cells were permeabilized with 0.5% Triton X-100 (Solarbio, Beijing, China) in PBS for 20 min, the cells were incubated with the Click-iT reaction mixture for 30 min in the dark. The nuclear DNA was counterstained with Hoechst 33342, and EdU-positive cells were visualized and recorded using an Olympus BX53E2C fluorescence microscope (Tokyo, Japan). For the MTT assay, cell proliferation was determined using the method described by Gong et al. [24]. L929 cells, HaCaT cells, and HUVECs were inoculated into 96-well plates (8 × 10^3^ cells/well) and cultured for 24 h. The culture medium was then replaced with various concentrations of AP10W (0, 0.5, 0.625, 0.75, 1, and 1.25 μg/mL). After an additional incubation period of 24 and 48 h, respectively, an aliquot of 20 μL MTT solution (5 mg/mL, Solarbio, Beijing, China) was then added to each well and the cells were further incubated for 4 h. The supernatant was then discarded, and 100 μL of dimethyl sulfoxide (DMSO) (Solarbio, Beijing, China) was added into each well to dissolve the generated formazan crystals. The optical density (OD) at 492 nm was detected by a microplate reader (Agilent BioTek Synergy H1, Santa Clara, CA, USA). The percentage of cell viability was calculated using the formula: Cell viability (%) = [(OD_t_ − OD_b_)/(OD_c_ − OD_b_)] × 100%, where OD_t_ represents the OD value of AP10W-treated cells, OD_b_ represents the OD value of the blank control, and OD_c_ represents the OD value of cells cultured in medium alone.

### 2.6. Assay for Tube Formation In Vitro

Angiogenesis was assessed using a tube formation assay. In short, following 12 h of starvation in FBS-free medium, HUVECs were harvested, and seeded into 96-well plates pre-coated with Matrigel^TM^ High Concentration Matrigel (Mogengel Bio, Xiamen, China) at the density of 5 × 10^4^ cells per well, in the presence of various concentrations of AP10W (0, 0.5, 0.75, 1, and 1.25 μg/mL), in accordance with previously established protocols [18]. After incubation at 37 °C for 4 h, an inverted phase contrast microscope (Nikon Eclipse Ts2, Tokyo, Japan) was used to quantify the total length of capillary-like networks and the number of network nodes in three random fields per culture plate well.

### 2.7. In Vitro Inflammation Model

Raw 264.7 cells were cultured in the absence or presence of 1 μg/mL lipopolysaccharide (LPS) (Solarbio, Beijing, China) for 12 h to induce inflammation. Subsequently, the cells were treated with 0.75 μg/mL AP10W for a further 12–24 h before harvest. The successful induction of inflammation and the effect of AP10W on macrophage polarization were evaluated via Western blotting and immunofluorescence staining to assess the expression of M1 markers cluster of differentiation 86 (CD86) and M2 markers CD206, as well as by quantitative real-time polymerase chain reaction (qRT-PCR) to detect the expression of the inflammatory (*Il-1β*, *Il-6*, *Tnf-α*, and *iNOS*) and anti-inflammatory (*Il-10* and *Arg-1*) factors.

### 2.8. Immunofluorescence Staining

Raw 264.7 cells treated as described in Section 2.7 were fixed in 4% PFA and permeabilized with 0.1% Triton X-100. Cells were then blocked with goat serum (Solarbio, Beijing, China) for 30 min at room temperature to prevent non-specific binding. The samples were subsequently incubated overnight at 4 °C with primary antibodies: mouse anti-CD86 (1:400; Santa Cruz Biotechnology, Dallas, TX, USA) and rabbit anti-CD206 (1:1000; Cell Signaling Technology, Danvers, MA, USA). After PBS washing, cells were incubated with secondary antibodies for 1 h at room temperature in the dark: Alexa Fluor^TM^488-conjugated donkey anti-mouse IgG (1:1000; Invitrogen, Carlsbad, CA, USA) and Alexa Fluor^TM^555-conjugated donkey anti-rabbit IgG (1:1000; Invitrogen, Carlsbad, CA, USA). Finally, nuclei were then counterstained with DAPI (Solarbio, Beijing, China). Stained cells were observed and imaged using an Olympus BX53E2C microscope (Olympus, Tokyo, Japan).

### 2.9. In Vivo Dorsal Skin Wound Model Assay

A total of forty male Kunming mice, aged 6–8 weeks, were purchased from Shandong Pengyue Experimental Animal Technology (ID: SCXK 20220006). The mice were housed in specific pathogen-free (SPF) conditions and maintained on a 12 h light/dark cycle (lights on: 08:00–20:00), with controlled temperature (25 ± 3 °C) and relative humidity (55 ± 10%). They were provided with a regular purified diet and water. As previously described [17], a dorsal skin wound model was established in mice. Briefly, anesthesia was induced by intraperitoneal injection of 2% sodium pentobarbital (Solarbio, Beijing, China). Dorsal hair was removed with an electric clipper and completely removed using depilatory cream, and the skin was cleaned with PBS. Full-thickness skin wounds with a diameter of 8 mm were created using a biopsy punch. Mice were then randomly divided into two groups (*n* = 20 per group): the control group and the AP10W group. Then, using a pipette, different drugs were administered dropwise onto the wound sites according to the assigned groups. Mice in the control group were administered 20 μL of sterile saline dropwise to each wound twice daily for 14 days, while those in the AP10W group received 20 μL of 20 μg/mL AP10W via the same administration protocol. The images of wounds were captured on days 0, 2, 4, 6, and 10 post wounding. Wounds were analyzed using ImageJ software (NIH, Bethesda, MD, USA). Wound margins were manually traced, and the wound area was calculated after calibration with a reference scale. The wound closure rate was determined as follows: wound closure (%) = [(initial wound area − residual wound area)/initial wound area] × 100%. Specimens of the wounds were harvested on days 3, 7, and 14 post wounding. Half of the tissues were fixed in 4% PFA for hematoxylin and eosin (H&E), Masson, immunofluorescence, and immunohistochemical staining. The other half of the tissues were stored in liquid nitrogen for Western blotting and qRT-PCR.

All animal experiments were performed in accordance with the guidelines of the Laboratory Animal Administration Ethics Committee of Shandong Second Medical University (Approval Code: 2024SDL521, Approval Date: 7 June 2024).

### 2.10. Assessment of Blood Flow in the Wound Area

On days 3 and 7 after surgery, RFLSI ZW Laser Speckle Blood Flow Imaging System (Model RFLSI ZW, RWD Life Science, Shenzhen, China) was used to assess microvascular network reconstruction in mice back wounds. The blood flow and the results were measured using the RFLSI Analysis software (V4.x).

### 2.11. H & E Staining, Masson Staining, Immunohistochemical, and Immunofluorescence Analysis

The fixed wound tissues were dehydrated, embedded in paraffin, and then sectioned into 5 μm thick slices. For histological evaluation, some sections were stained with an H&E staining kit (Solarbio, Beijing, China) and a Masson staining kit (Solarbio, Beijing, China) to observe the histological structure and collagen composition of wound tissues, respectively. For immunohistochemical staining, paraffin sections were first blocked with bovine serum albumin (Solarbio, Beijing, China) at room temperature for 1 h to prevent non-specific binding. Subsequently, the sections were incubated overnight at 4 °C with primary antibodies: rabbit anti-VEGFA (1:200; Proteintech, Rosemont, IL, USA), mouse anti-CD86 (1:400; Santa Cruz Biotechnology), and rabbit anti-CD206 (1:500; Cell Signaling Technology). After washing with PBS, the sections were incubated with horseradish peroxidase (HRP)-conjugated secondary antibodies (1:200; ImmunoWay, Plano, TX, USA) for 1 h at room temperature. Finally, the sections were stained with a 3,3′-diaminobenzidine (DAB) substrate kit (Beyotime, Shanghai, China), followed by hematoxylin counterstaining of the nuclei (Solarbio, Beijing, China). For immunofluorescence staining, paraffin sections were incubated overnight at 4 °C with the indicated primary antibodies: rat anti-CD31 (1:200; Santa Cruz Biotechnology), rabbit anti-α-SMA (1:200; ABmart, Shanghai, China) [18], rabbit anti-PCNA (1:200; Proteintech, Rosemont, IL, USA) and rabbit anti-YAP (1:200; Proteintech, Rosemont, IL, USA). Following PBS washing, sections were incubated with Alexa Fluor^TM^546-conjugated goat anti-rat secondary antibody (1:400; Invitrogen, Carlsbad, CA, USA), FITC-labeled goat anti-rabbit secondary antibody (1:400; Beyotime, Shanghai, China) and Alexa Fluor^TM^555-conjugated donkey anti-rabbit secondary antibody (1:400; Invitrogen, Carlsbad, CA, USA) for 1 h at room temperature. Finally, the nuclei were counterstained with DAPI (Solarbio, Beijing, China). All stained sections were observed and imaged using an Olympus BX53E2C microscope (Olympus, Tokyo, Japan). For quantitative analysis, 3–5 random fields of view were selected per tissue section based on the size of the granulation tissue, and image analysis was performed using ImageJ software (NIH).

### 2.12. Western Blotting Analysis

HUVECs were seeded in a 60 mm culture dish and cultured at 37 °C. When cell confluence reached approximately 60%, the medium was replaced with serum-free medium for overnight starvation, after which the cells were exposed to 1 μg/mL AP10W for 24 h. The treated cells were subsequently collected and lysed on ice for 30 min using lysis buffer (20 mM Tris pH 7.5, 150 mM NaCl, 1% Triton X-100) containing protease and phosphatase inhibitor cocktail (1×) (Beyotime, Shanghai, China) at 4 °C for 30 min. The lysates were centrifugated at 12,000× *g* for 15 min at 4 °C, and the supernatants were pooled. For the wound skin tissues obtained as described above, they were homogenized in 1× PBS (pH 7.4) containing protease and phosphatase inhibitor cocktail using a Polytron homogenizer and sonicator. The homogenates were centrifuged at 12,000× *g* for 15 min at 4 °C, and the supernatants were pooled. Protein concentration was detected using a BCA Protein Assay Kit (Beyotime, Shanghai, China). Equivalent amounts of total protein (40 μg) were electrophoresed on 10–15% sodium dodecyl sulfate-polyacrylamide gel electrophoresis and then transferred onto polyvinylidene fluoride (PVDF) membranes (Millipore, Burlington, MA, USA). The membranes were blocked with 5% BSA in PBST at room temperature for 2 h and then incubated overnight at 4 °C with the primary antibodies against PCNA (1:1000; Proteintech, Rosemont, IL, USA), YAP (1:1000; Proteintech Rosemont, IL, USA), phospho-YAP (1:1000; Bioworlde, Louis Park, MN, USA). Following three washes with PBST, the membranes were incubated with corresponding HRP-conjugated secondary antibodies (1:5000, Proteintech Group, Inc., Rosemont, IL, USA) for 1 h at room temperature. The membranes were then incubated with an ECL kit (Epizyme, Shanghai, China) and imaged with a Mini Chemifluorescence imaging analysis system (Clinx Science Instruments Co., Ltd., Shanghai, China). The band intensity was analyzed using ImageJ software (NIH, Bethesda, MD, USA), quantified and normalized to GAPDH.

### 2.13. RNA Isolation and Quantitative Real-Time Polymerase Chain Reaction (qRT-PCR)

Total RNA was isolated from cells by TRIzol (TIANGEN, Beijing, China) according to the instructions provided by the reagent manufacturer. Reverse transcription was performed by application of Hifair^®^ AdvanceFast one-step RT-gDNA Digestion SuperMix for qPCR (Yeasen, Shanghai, China) according to the manufacturer’s instructions. The resulting cDNA was stored at −20 °C until use. qRT-PCR was carried out using 2× M5 HiPer SYBR Premix EsTaq (Mei5bio, Beijing, China) on an Applied Biosystems QuantStudio™ 5 Real-Time PCR Instrument (96-Well 0.2 mL Block) (Thermo Fisher Scientific, Waltham, MA, USA) according to the protocol provided by the manufacturer. The sequences of primers were listed in Table 1 and Table 2. Human *GAPDH* or mouse *Gapdh* was selected as the internal control, as appropriate. Gene expression levels were normalized to the internal reference gene and assessed using the 2^−ΔΔCT^ method.

### 2.14. Statistical Analysis

The experimental data were expressed as mean ± SD or mean ± SEM and analyzed using GraphPad Prism Software (Version 9.0, GraphPad Software, Inc., San Diego, CA, USA). Statistical differences were evaluated using Student’s *t*-test or one-way ANOVA. *p* values of less than 0.05 were considered a significant difference (* *p* < 0.05, ** *p* < 0.01 and *** *p* < 0.001 indicate a statistically significant difference compared with the control group; # *p* < 0.05, ## *p* < 0.01 and ### *p* < 0.001 indicate a statistically significant difference compared with the model group). All in vitro experiments were performed with at least three independent biological replicates. For in vivo experiments, the sample size for each group is indicated in the corresponding figure legends.

The schematic figures in this study were created by the authors using BioRender (https://www.biorender.com/; accessed on 9 April 2026).

## 3. Results

### 3.1. AP10W Promoted Migration and Proliferation of Fibroblasts and Keratinocytes In Vitro

**Figure 1:**
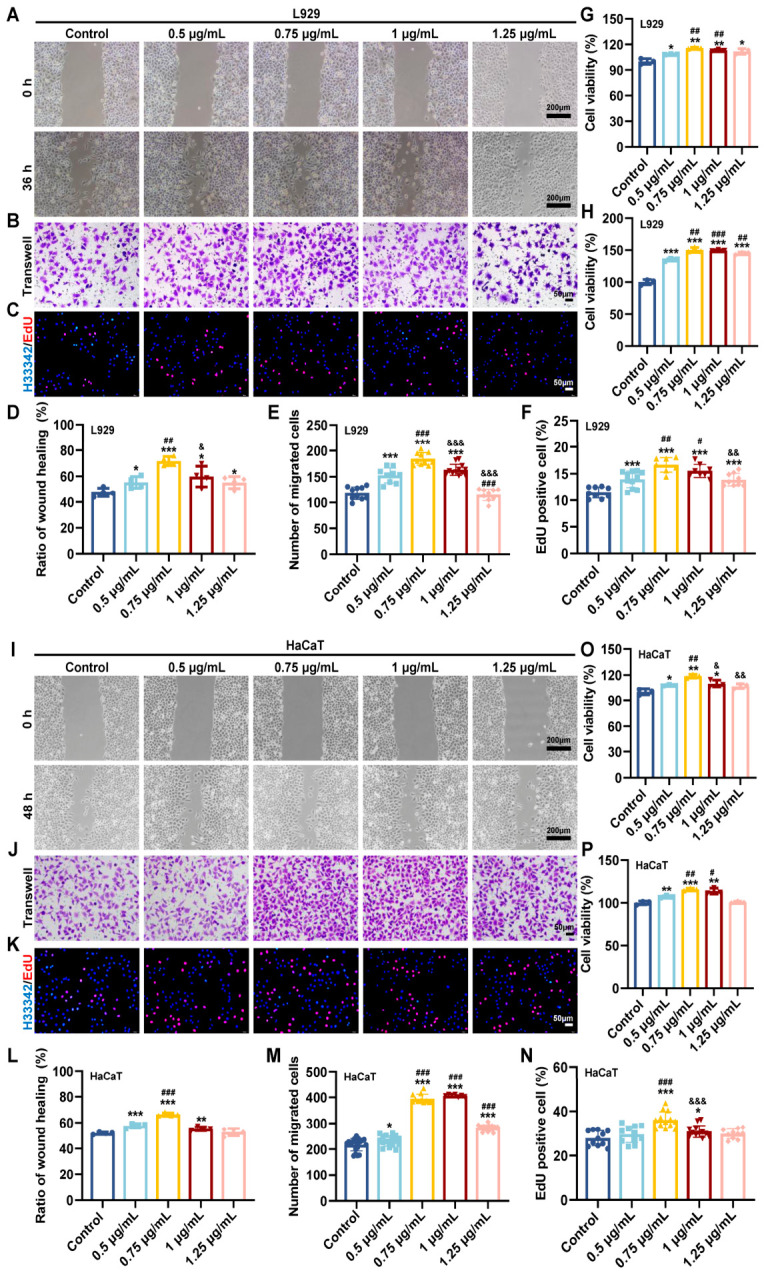
AP10W promotes the migration and proliferation of fibroblasts and keratinocytes in vitro. (**A**,**D**) L929 fibroblasts were treated with different concentrations of AP10W. Representative images and quantitative analysis of cell migration in scratch wound healing assays are shown (*n* = 3). Scale bar = 200 μm. (**B**,**E**) Representative images and quantitative analysis of migrated L929 fibroblasts in transwell assays are shown (*n* = 3). Scale bar = 50 μm. (**C**,**F**) EdU incorporation assays were performed to evaluate the effect of AP10W on L929 fibroblast proliferation. Nuclei were counterstained with Hoechst 33342 (*n* = 10 microscope fields). Scale bar = 50 μm. (**G**,**H**) Cell proliferation of L929 fibroblasts was determined using the MTT assay (*n* = 3). (**I**,**L**) HaCaT keratinocytes were treated with different concentrations of AP10W. Representative images and quantitative analysis of cell migration in scratch wound healing assays are shown (*n* = 3). Scale bar = 200 μm. (**J**,**M**) Representative images and quantitative analysis of migrated HaCaT keratinocytes in transwell assays are shown (*n* = 3). Scale bar = 50 μm. (**K**,**N**) EdU incorporation assays were performed to evaluate the effect of AP10W on HaCaT keratinocyte proliferation. Nuclei were counterstained with Hoechst 33342 (*n* = 10 microscope fields). Scale bar = 50 μm. (**O**,**P**) Cell proliferation of HaCaT keratinocytes was determined using the MTT assay (*n* = 3). Data are shown as mean ± SD, * *p* < 0.05, ** *p* < 0.01, and *** *p* < 0.001 vs. control group; # *p* < 0.05, ## *p* < 0.01, and ### *p* < 0.001 compared with AP10W (0.5 µg/mL)-treated group; and ^&^ *p* < 0.05, ^&&^ *p* < 0.01, and ^&&&^ *p* < 0.001 compared with AP10W (0.75 µg/mL)-treated group.

To explore the biological significance of AP10W in wound healing, we selected two types of skin repair cells (fibroblasts and keratinocytes), which are very important in wound healing [29]. The migration of these cells is a fundamental cellular mechanism driving wound re-epithelialization and closure [30]. We first assessed the effect of AP10W on cell migration using scratch wound healing and transwell assays. In scratch assays, AP10W significantly enhanced the wound closure rate of both L929 and HaCaT cells in a time- and dose-dependent fashion (Figure 1A,I). The most pronounced effect was observed at 0.75 μg/mL. For L929 cells, AP10W (0.75 μg/mL) increased the wound healing rate to 71.76% at 36 h, which was 50.61% higher than the control group (Figure 1D). Similarly, in HaCaT cells, the healing rate increased to 66.39 ± 1.24% with AP10W (0.75 μg/mL) compared to 51.89 ± 0.84% in the control after 48 h (Figure 1L). Consistent with these observations, transwell assays confirmed the pro-migratory effect of AP10W. Treatment with AP10W (0.5–1.25 μg/mL) significantly increased the number of migrated L929 and HaCaT cells across the membrane (Figure 1B,J). Quantitative analysis showed that the number of migrated L929 cells peaked at 184.20 ± 11.75 (Figure 1E), while HaCaT cell migration peaked at 406.14 ± 4.18 (Figure 1M) at 0.75 μg/mL and 1 μg/mL, respectively. These results demonstrate that AP10W promotes the migration of both fibroblasts and keratinocytes, which is consistent with previous reports on other AMPs [17,27].

In addition to migration, cell proliferation is equally crucial for effective tissue repair [29]. We therefore evaluated the impact of AP10W on the proliferative capacity of L929 and HaCaT cells using EdU incorporation and MTT assays. The EdU assay revealed a significant increase in the proportion of EdU-positive cells following AP10W treatment (*p* < 0.05, Figure 1C,K). The percentage of EdU-positive L929 cells increased from 11.47 ± 1.04% (control) to 16.65 ± 1.34% (0.75 μg/mL), and HaCaT cells increased from 27.86 ± 3.42% to 36.09 ± 3.64% (Figure 1F,N). Supporting these observations, the results of the MTT assay also indicated that AP10W (0.5–1.25 μg/mL) markedly enhanced the viability of both cell lines (Figure 1G,H,O,P) after 24 h and 48 h of treatment. Collectively, these findings indicate that AP10W plays an important functional role in wound healing by stimulating the key process of migration and proliferation in both fibroblasts and keratinocytes.

### 3.2. AP10W Promoted Endothelial Cell Proliferation, Migration, and Angiogenic Activity

**Figure 2:**
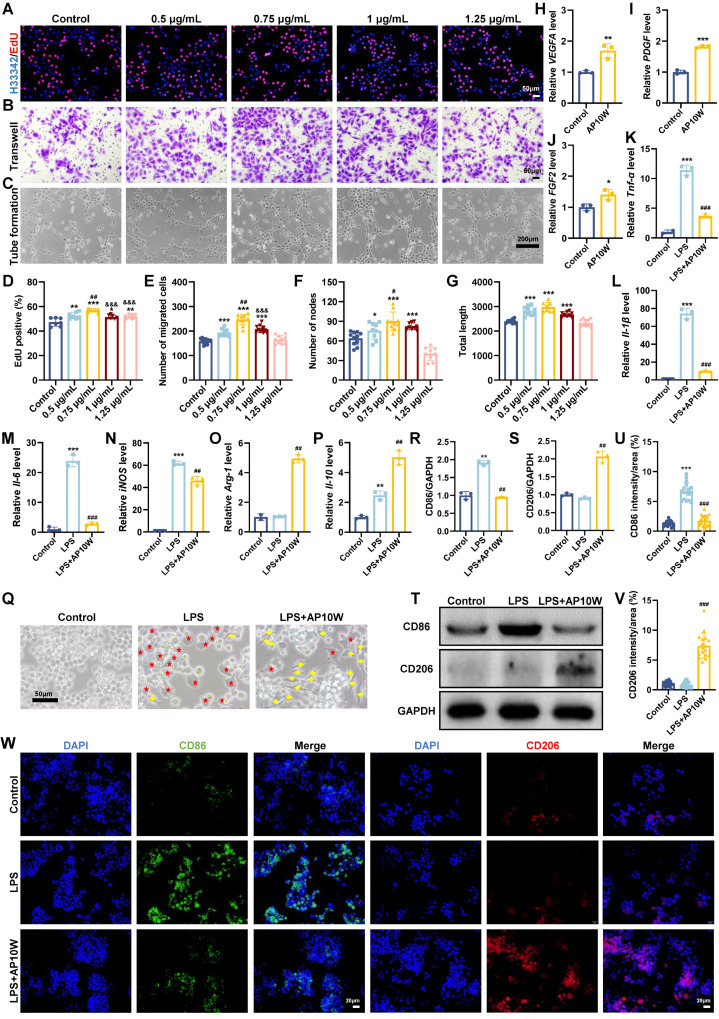
AP10W enhances endothelial cell proliferation, migration, and tube formation, and promotes macrophage polarization from M1 to M2 phenotype in vitro. (**A**,**D**) EdU incorporation assays were performed to evaluate the effect of AP10W on HUVEC proliferation. Nuclei were counterstained with Hoechst 33342 (*n* = 10 microscope fields). Scale bar = 50 μm. (**B**,**E**) Representative images and quantitative analysis of migrated HUVECs in transwell assays are shown (*n* = 3). Scale bar = 50 μm. (**C**,**F**,**G**) The angiogenic effect of AP10W was evaluated using a tube formation assay. Representative images of tube-like structures, and quantitative analysis of the number of nodes and total length are shown (*n* = 3). Scale bar = 200 μm. Data are shown as mean ± SD, * *p* < 0.05, and *** *p* < 0.001 vs. control group; # *p* < 0.05 compared with AP10W (0.5 µg/mL)-treated group. (**H**–**J**) qRT-PCR analysis of *VEGFA*, *PDGF*, *FGF2* mRNA expression in HUVECs (*n* = 3). (**K**–**P**) qRT-PCR analysis of *Tnf-α*, *Il-1β*, *Il-6*, *iNOS*, and *Arg-1*, *Il-10* mRNA expression in Raw 264.7 cells (*n* = 3). (**Q**) Representative morphology of macrophages cultured under different conditions. Red asterisks and yellow squares indicate M1 and M2 macrophages, respectively. Scale bar = 50 μm. (**T**) Western blotting analysis of CD86 and CD206 protein expression. (**R**,**S**) Quantitative analysis of CD86 and CD206 protein expression in Raw 264.7 cells after 24 h of treatment (*n* = 3). (**U**–**W**) Representative immunofluorescence images and quantitative analysis of Raw 264.7 cells stained for the M1 biomarker CD86 (green) and the M2 biomarker CD206 (red), respectively (*n* = 3). Scale bar = 20 μm. Data are shown as mean ± SD, ** *p* < 0.01 and *** *p* < 0.001 vs. control group, and ## *p* < 0.01 and ### *p* < 0.001 vs. LPS group; ^&&&^ *p* < 0.001 compared with AP10W (0.75 µg/mL)-treated group.

Angiogenesis is a critical, multistep process for wound healing, involving the proliferation, migration, and lumen formation of endothelial cells to generate new blood vessels [31]. To evaluate the pro-angiogenic potential of AP10W, we assessed its effects on these key cellular functions in HUVECs. First, an EdU incorporation assay demonstrated that AP10W (0.5–1.25 μg/mL) significantly enhanced the proliferation of HUVECs in a dose-dependent manner (Figure 2A). The most potent effect was observed at 0.75 μg/mL, where the proportion of EdU-positive cells increased to 56.97 ± 1.44%, compared to 47.32 ± 3.41% in the control group (Figure 2D). Next, a transwell assay revealed that AP10W (0.5–1.25 μg/mL) markedly stimulated the migratory capacity of HUVECs (Figure 2B). The effect peaked at 0.75 μg/mL, with the number of migrated cells reaching 246.70 ± 21.50, significantly higher than the 159.27 ± 10.62% observed in the control group (*p* < 0.001, Figure 2E). Finally, to determine the functional outcome of increased proliferation and migration, we performed a tube formation assay. Treatment with AP10W led to a pronounced enhancement in angiogenic activity, evidenced by a marked increase in both the total capillary length and the number of nodes compared to the control (Figure 2C,F,G). These suggest that AP10W have the capacity to induce tube formation in endothelial cells. Therefore, we next examined the effect of AP10W on the expression of key angiogenic growth factors in HUVECs at a concentration of 0.75 μg/mL by qRT-PCR assay. It showed that treatment with AP10W upregulated the expression of *VEGFA*, *PDGF*, and *FGF2* (Figure 2H–J). In summary, all of the aforementioned data indicate that AP10W promotes key steps of angiogenesis by stimulating endothelial cell proliferation, migration, and tube formation.

### 3.3. AP10W Promotes Macrophage Polarization from M1 to M2 In Vitro

Macrophages are the key immune cells that orchestrate wound healing progress, and their functional plasticity is categorized into major subsets: classically activated (M1) macrophages, implicated in pro-inflammatory events, and alternative-activated (M2) macrophages, thought to be anti-inflammatory and pro-regenerative [6,32]. In chronic wounds, the transformation of macrophages from M1 to M2 phenotype is frequently impaired, resulting in persistent inflammation and failure to progress into the proliferative phase of repair [33]. To investigate whether AP10W influences macrophage polarization, we established an in vitro inflammatory model by stimulating Raw 264.7 macrophages with LPS. Successful M1 polarization was confirmed by increased protein expression of the M1 marker CD86 as assessed by Western blotting and immunofluorescence staining (Figure 2R–W). Notably, treatment with AP10W significantly reversed this LPS-induced polarization. Immunofluorescence analysis revealed that AP10W treatment markedly reduced CD86 expression (green) while enhancing CD206 expression (red) in LPS-stimulated macrophages compared to the LPS-only group (Figure 2W). Consistent with these observations, Western blotting confirmed a significant decrease in CD86 protein levels and a concomitant increase in CD206 levels following AP10W treatment (Figure 2T). At the transcriptional level, qRT-PCR analysis demonstrated that AP10W significantly downregulated the expression of M1-associated genes, including *Tnf-α*, *Il-1β*, *Il-6*, and *iNOS* (Figure 2K–N). Conversely, the expression of M2-associated genes, such as *Arg-1* and *Il-10*, was markedly upregulated (Figure 2O,P). Furthermore, morphological examination revealed that AP10W treatment induced a phenotypic shift in LPS-stimulated macrophages, with cells acquiring a more elongated, M2-like morphology (Figure 2Q). Collectively, these findings demonstrate that AP10W promotes the functional reprogramming of macrophages from a pro-inflammatory M1 phenotype toward a pro-healing M2 phenotype, thereby contributing to the resolution of inflammation and facilitating tissue repair.

### 3.4. AP10W Accelerates Wound Healing in Vivo

**Figure 3:**
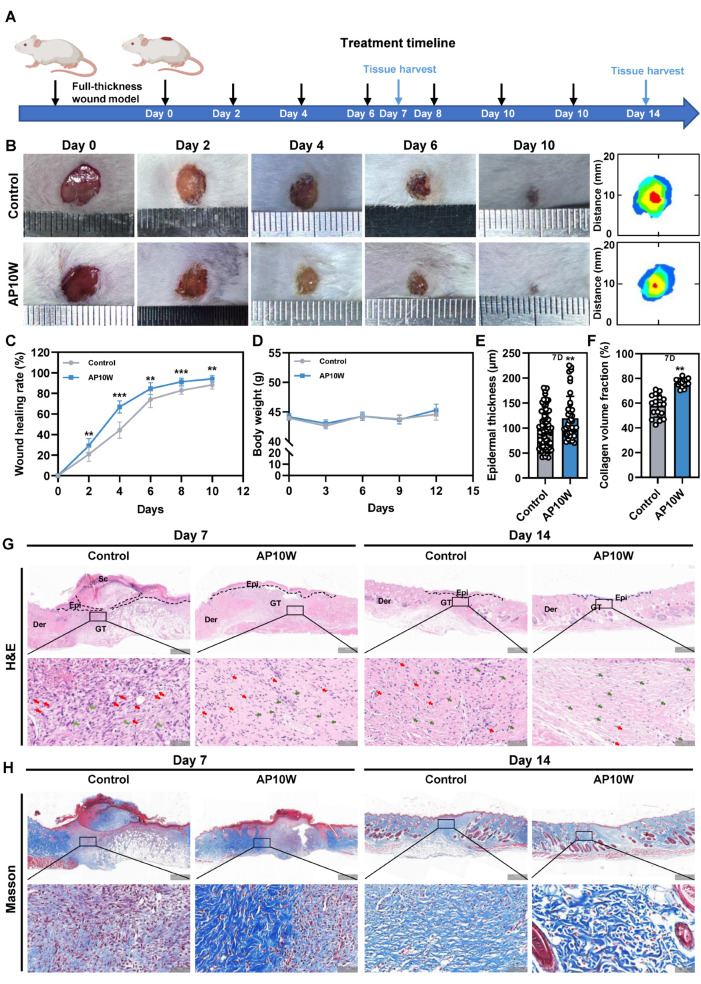
AP10W accelerates wound healing in a murine full-thickness wound model. (**A**) Schematic illustration of the murine full-thickness wound model and the treatment regimen during the healing process. (**B**) Representative images of wounds at different time points post-wounding, along with merged wound closure traces for each group. (**C**) Quantitative analysis of wound closure rates within 10 days post-wounding in different groups (*n* = 10). (**D**) Body weight of mice in different groups at indicated time points. Data are presented as the mean ± SEM (*n* = 5). (**E**) Relative epithelial thickness at the wound site on day 7 (*n* = 3). (**F**) Relative collagen content at the wound site on day 7 (*n* = 3). (**G**) Representative H & E-stained images of wound tissues from different groups on days 7 and 14 (*n* = 3 per time points). Black dotted lines indicate newly formed tissues. Sc, scab; Epi, epidermis; Der: dermis; and GT, granulation tissue. Scale bar = 500 μm. Insets show magnified views of the box areas (red arrows, inflammatory cells; green arrows, fibroblasts). Scale bar = 50 μm. (**H**) Representative Masson’s trichrome stained-images of wound tissues. Scale bar = 500 μm. Insets show magnified views of the boxed areas. Scale bar = 50 μm. Data are shown as mean ± SD, ** *p* < 0.01 and *** *p* < 0.001 vs. control group.

To evaluate whether AP10W can enhance wound closure in vivo, full-thickness excision wounds were created on the dorsal skin of mice with a diameter of 8 mm. The wounds were treated with either 20 μL of AP10W (20 μg/mL) or an equal volume of sterile saline (control) twice daily (Figure 3A). Digital photographs of wounds revealed that much faster wound closure was found when exposed to AP10W, as determined by consistently smaller wound areas measured on days 2, 4, 6, 8, and 10 post-wounding compared with control group (Figure 3B,C). The wound closure rate reached 66.83% by day 4 and 94.20% by day 10 in the AP10W-treated mice, both rates being significantly higher than those in the control group (*p* < 0.01; Figure 3C). No death or abnormality was observed in any animal during the postoperative period (Figure 3D).

Histological analysis was performed to elucidate the mechanisms underlying the accelerated closure. H&E staining on day 7 post-wounding showed that administration of AP10W significantly promoted re-epithelialization, a critical early healing phase. This was evidenced by a longer neo-epithelial tongue and increased epidermal thickness compared to the control group (Figure 3E,G). By day 14, the epidermal thickness in AP10W-treated wounds had normalized and was remarkably thinner than the still hyperplastic epithelium of control wounds (Figure 3G and Figure S1). This transition from a thick, proliferative epithelium to a mature, thin layer indicates progression beyond the inflammatory phase and completion of re-epithelialization [34]. Furthermore, by day 14, AP10W-treated wounds exhibited advanced regeneration, including the reappearance of mature skin structures such as hair follicles and sebaceous glands, which were less in controls (Figure 3G). H&E staining also indicated enhanced tissue remodeling, characterized by reduced inflammatory cell infiltration (red arrows) and an increased fibroblast population (green arrows) in the AP10W group on days 7 and 14 (Figure 3G). Subsequently, Masson’s trichrome staining was used to assess collagen deposition. Consistent with the improved epidermal healing, AP10W-treated wounds displayed denser, thicker, and more regularly organized collagen fibers compared to controls on both days 7 and 14 (Figure 3F,H). All of the aforementioned data suggest that topical administration of AP10W potently accelerates wound healing in vivo by enhancing re-epithelialization, promoting granulation tissue formation and maturation, and facilitating the regeneration of skin appendages.

### 3.5. AP10W Accelerates Wound Healing by Enhancing Angiogenesis and Proliferation, and Re-Programming M1 to M2 In Vivo

**Figure 4:**
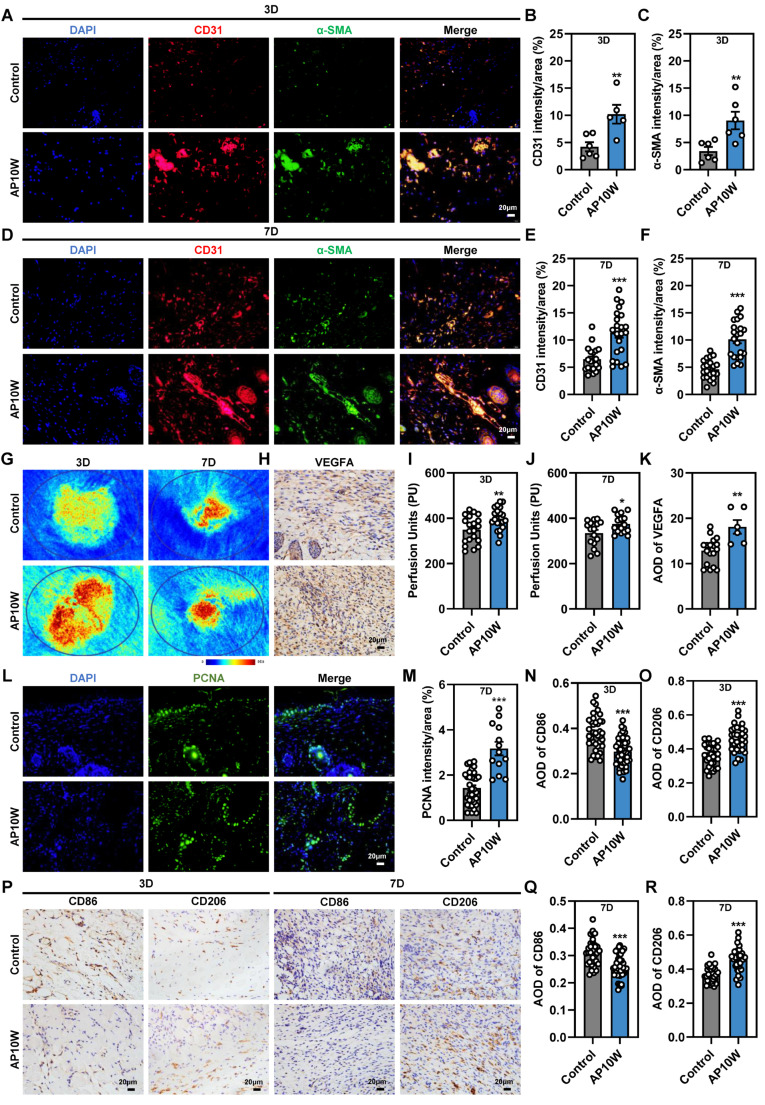
AP10W accelerates wound healing by enhancing angiogenesis and proliferation, and by promoting the transition from the M1 to the M2 macrophage phenotype in vivo. (**A**–**F**) Representative images and quantitative analysis of CD31^+^ (red) and α-SMA^+^ (green) vessels in wound tissues on days 3 and 7 in the mice treated with AP10W (20 µg/mL) or sterile saline (control). Nuclei were counterstained with DAPI (blue) (*n* = 3). Scale bar: 20 µm. (**G**) Representative laser Doppler perfusion images of wounds in different groups. (**H**) Representative immunohistochemical images of VEGFA-positive cells (brown) in wound tissues on day 7. Scale bar: 20 µm. (**I**,**J**) Quantitative analysis of perfusion signals. In the color-coded images, the highest perfusion values are shown in red, intermediate values in yellow to light blue, and the lowest values in dark blue. (**K**) Quantitative analysis of VEGFA^+^ cells (*n* = 3). (**L**,**M**) Representative images and quantitative analysis of PCNA-positive cells (green) in wound tissues on day 7. Nuclei were stained with DAPI (blue) (*n* = 3). Scale bar: 20 µm. (**N**,**O**) Quantitative analysis of CD86^+^ and CD206^+^ cells on day 3, respectively (*n* = 3). (**P**) Representative immunohistochemical images of CD86^+^ or CD206^+^ cells (brown) in wound tissues on days 3 and 7. Scale bar: 20 µm. (**Q**,**R**) Quantitative analysis of CD86^+^ or CD206^+^ cells on day 7, respectively (*n* = 3). Data are presented as the mean ± SEM. * *p* < 0.05, ** *p* < 0.01 and *** *p* < 0.001 vs. control group.

To explore the potential mechanism by which the AP10W promotes healing, we analyzed key biomarkers on days 3 and 7 after injury. First, in order to evaluate the formation of new vessels at the wound sites, angiogenesis-related growth factors were analyzed by immunofluorescence staining of CD31 and α-SMA, immunohistochemical staining of VEGF, and local animal Doppler detection (Figure 4A–K). CD31 is a marker to represent the vascular endothelial cells, while α-SMA is the actin isoform typical of smooth muscle cells to show the vascular wall [34,35]. CD31 immunofluorescence staining revealed a remarkably dense population of red CD31-positive cells (red fluorescence) in the AP10W group compared to that in the control group on days 3 and 7 post-wounding, indicating that dense microvascular structures were formed in the wounds of the AP10W group (Figure 4A–F). Moreover, the green fluorescence of α-SMA, which represents neonatally mature blood vessels, is more pronounced in the AP10W group on both 3 and 7 days, confirming that AP10W was able to promote angiogenesis in wound repair (Figure 4A–F). Quantitative analysis showed that the expression of CD31 and α-SMA in the AP10W group was increased 1.76 fold and 2.19 fold, respectively, by day 7 (Figure 4E,F). Consistent with this, local animal Doppler imaging demonstrated significantly higher blood perfusion units (PUs) in the AP10W group on days 3 and 7 (Figure 4G,I,J). Furthermore, immunohistochemical staining confirmed that AP10W treatment markedly upregulated the expression of key pro-angiogenic factor VEGFA in the wound bed (Figure 4H,K). Next, we assessed the effect of AP10W on the macrophage polarization using the specific markers CD86 (M1) and CD206 (M2). Immunohistochemical analysis showed that control wounds on days 3 and 7 maintained a relatively high level of CD86^+^ M1 macrophages and a low level of CD206^+^ M2 macrophages, indicative of a prolonged inflammatory state (Figure 4N–R). In contrast, AP10W-treated wounds exhibited a significant shift toward a pro-healing phenotype, characterized by reduced CD86^+^ M1 macrophages and a concomitant increase in CD206^+^ M2 macrophages (Figure 4N–R). This reprogramming from a pro-inflammatory to a pro-reparative macrophage profile aligns with the observed histological transition out of the inflammatory phase. Finally, staining for proliferation marker PCNA indicated a considerably higher number of proliferating cells in the wound areas of AP10W-treated mice compared to controls (Figure 4L,M), consistent with our in vitro findings on fibroblasts and keratinocytes. In summary, AP10W accelerates wound repair in vivo through a multiple mechanism that includes promoting angiogenesis, enhancing cellular proliferation, and facilitating the beneficial reprogramming of wound macrophages from an M1 to an M2 phenotype.

### 3.6. AP10W Accelerates Wound Healing by Activating YAP Signaling

**Figure 5:**
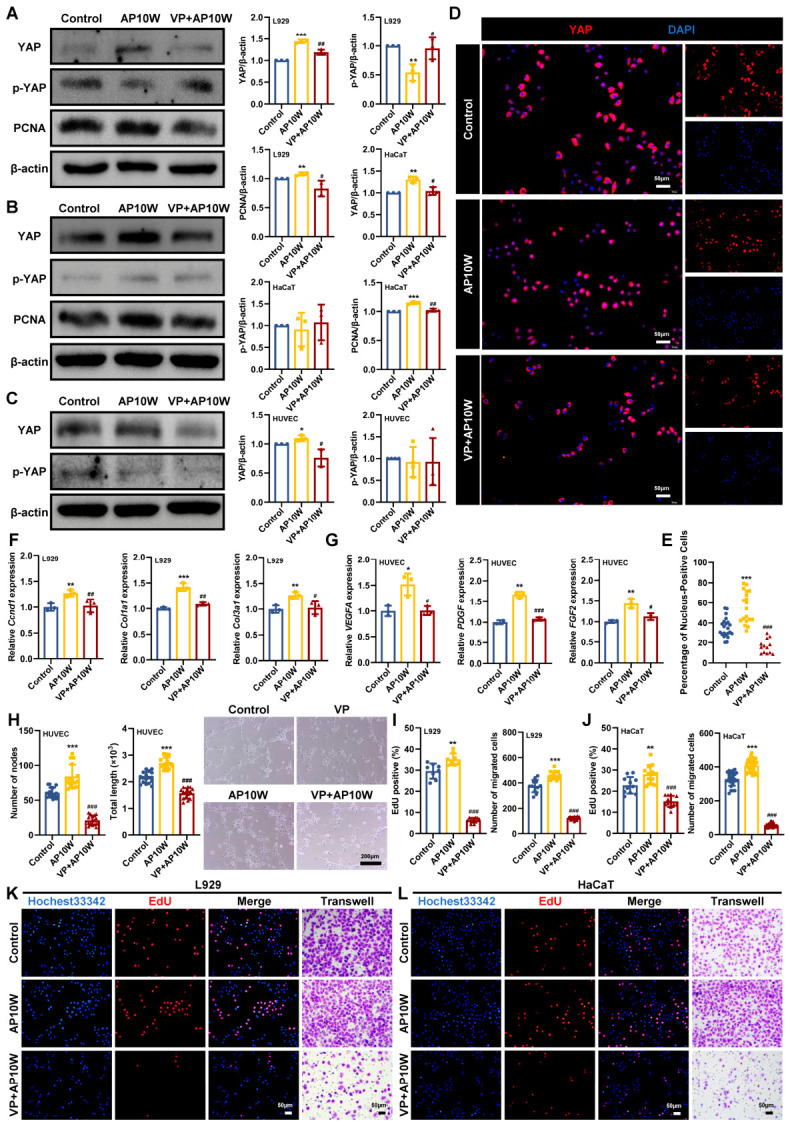
AP10W accelerates wound healing by activating YAP signaling. (**A**–**C**) Western blotting analysis of YAP, p-YAP, and PCNA protein expression in L929, HaCaT, and HUVECs, respectively (*n* = 3). (**D**,**E**) Representative immunofluorescence of YAP localization (red) in L929 cells. Nuclei were counterstained with DAPI (blue) (*n* = 3). Scale bar: 50 µm. (**F**) qRT-PCR analysis of *Ccnd1*, *Col1a1*, and *Col3a1* mRNA expression in L929 cells (*n* = 3). (**G**) qRT-PCR analysis of *VEGFA*, *PDGF*, and *FGF2* mRNA expression in HUVECs (*n* = 3). (**H**) Representative images of tube formation and quantitative analysis of the number of nodes and total length in HUVECs (*n* = 3). Scale bar: 200 µm. (**I**–**L**) Representative images and quantitative analysis of EdU incorporation in L929 and HaCaT cells, respectively (*n* = 3). Scale bar: 50 µm. Data are presented as mean ± SD, * *p* < 0.05, ** *p* < 0.01, and *** *p* < 0.001 vs. control group; # *p* < 0.05, ## *p* < 0.01 and ### *p* < 0.001 vs. AP10W group.

YAP is a key transcriptional coactivator that regulates cell behavior, and its movement into the nucleus is known to be critical for efficient skin wound healing [19]. To explore whether the pro-healing effects of AP10W are related to YAP signaling, we first examined the expression of YAP and phosphorylated YAP (p-YAP) in L929, HaCaT, and HUVECs (Figure 5A–C). Western blotting analysis showed that AP10W treatment clearly increased YAP expression in all three cell types compared with the control group. At the same time, p-YAP levels were significantly reduced in L929 cells, whereas few differences were observed in HaCaT and HUVECs. We then used immunofluorescence staining to further assess YAP localization. As shown in Figure 5D,E, AP10W markedly promoted YAP nuclear translocation. To further confirm whether YAP is functionally involved in the pro-healing effects of AP10W, we used verteporfin (VP), a well-characterized small molecular YAP inhibitor [21]. Notably, pretreatment with VP (VP + AP10W) significantly reduced YAP expression and weakened its nuclear translocation (Figure 5A–E). This inhibitory effect was accompanied by a clear loss of AP10W-induced biological activity. Specially, YAP inhibition reduced PCNA expression (Figure 5A,B), decreased EdU incorporation, lowered the number of migrated cells (Figure 5I–L), and impaired angiogenic activity in HUVECs, as reflected by reduced total capillary length and fewer nodes (Figure 5H). At the gene level, qRT-PCR analysis further showed that blocking YAP significantly weakened the AP10W-induced upregulation of genes related to cell cycle progression (*Ccnd1*), extracellular matrix remodeling (*Col1a1* and *Col3a1*), and angiogenesis (*VEGFA*, *PDGF* and *FGF2*) (Figure 5F,G). Overall, these findings indicate that AP10W promotes wound repair, at least in part, by activating the YAP signaling pathway.

## 4. Discussion

Although the general cellular and molecular events underlying cutaneous wound healing have been well characterized [36,37], impaired wound healing remains a significant clinical challenge [7]. Wounds that fail to progress through the normal healing sequence are often characterized by multiple coexisting pathological factors, including persistent inflammation, microbial infection, impaired angiogenesis, and insufficient vascular perfusion, which mutually reinforce each other [38,39]. Therefore, therapeutic agents capable of targeting several of these defects simultaneously may provide greater benefit than strategies focused solely on antimicrobial activity or single-pathway modulation [39]. In this context, antimicrobial peptides have emerged as promising multifunctional molecules [2,8]; however, whether AP10W possesses direct tissue-reparative and immunomodulatory activities beyond its known broad-spectrum antimicrobial and antifungal effects [24,25] remains unclear. Thus, the present study aimed to systematically evaluate the pro-healing potential of AP10W and to elucidate its effects on key wound-healing events, including keratinocyte and fibroblast migration, endothelial angiogenic responses, macrophage polarization, and the underlying signaling mechanism.

AMPs have attracted increasing attention as multifunctional therapeutic candidates for chronic wound management [8,40]. In the present study, we identify AP10W as a multifunctional AMP with intrinsic pro-healing properties in addition to its previously confirmed broad-spectrum antimicrobial and antifungal activities [24,25]. Unlike therapeutic approaches that rely primarily on reducing microbial burden, AP10W directly enhanced several core regenerative processes in vitro, including the migration and proliferation of fibroblasts, keratinocytes, and endothelial cells, while also promoting macrophage polarization toward a pro-reparative M2 phenotype. These cellular effects were further confirmed in vivo, where topical AP10W treatment accelerated wound closure, enhanced re-epithelialization, promoted granulation tissue maturation, improved collagen deposition, and facilitated the regeneration of skin appendages. Taken together, these findings suggest that AP10W exerts coordinated actions across multiple stages of wound healing and further support the concept that AMPs function not only as anti-infective molecules but also as active modulators of tissue repair.

A hallmark of effective wound healing is the rapid restoration of epidermal continuity together with reconstruction of the dermal matrix [37]. In the present study, AP10W clearly promoted structural repair in vivo, as evidenced by reduced inflammatory cell infiltration, an increased fibroblast population, a longer neo-epithelial tongue, more prominent reappearance of mature skin structures, and denser, thicker, and more regularly organized collagen fibers in AP10W-treated wounds. Notably, AP10W-treated wounds exhibited a transition from a thick, proliferative epithelium to a mature and thin epidermal layer, which is generally considered indicative of progression beyond the inflammatory phase and completion of re-epithelialization [34]. These histological observations were consistent with our in vitro observations that AP10W enhanced the proliferation and migration of both keratinocytes and fibroblasts. This is biologically relevant because keratinocyte motility is essential for re-epithelialization, whereas fibroblast expansion and activation drive granulation tissue formation, collagen deposition, and tissue remodeling [2]. In this respect, our data are in line with previous reports showing that several AMPs, including LL-37, hBDs, Histatin 1 and 2, AMP-IBP5, S100A7 and S100A15, HNP-1, Brevinin-2PN, PM-7, TP2-5 and TP2-6 play active roles in promoting collagen synthesis and facilitating tissue remodeling [18]. Similarly, AMP-IBP5 has been shown to enhance keratinocyte and fibroblast responses [16], further supporting the view that AP10W belongs to a growing class of AMPs with direct tissue reparative properties.

In addition to structural restoration, proper control of the inflammatory response is indispensable for successful healing. Among immune cells involved in wound repair, macrophages play a pivotal role by orchestrating the transition from inflammation to tissue regeneration and by dynamically adopting distinct phenotypes in response to the wound microenvironment [41]. Classically activated M1 macrophages produce inflammatory mediators, including nitric oxide, ROS, IL-1β, IL-6, and TNF-α, thereby amplifying the inflammatory response [42]. As healing progresses, macrophages normally shift toward the M2 phenotype, which is associated with inflammation resolution, tissue repair, and neovascularization through the secretion of factors such as IL-10, VEGF, and TGF-β [43]. In impaired wound healing, however, this M1 to M2 transition is often dysregulated, resulting in persistent inflammation and delayed progression into the reparative phase [44]. Notably, previous studies have shown that different AMPs can regulate macrophage polarization in distinct ways to support wound healing. For example, LL-37 promotes polarization toward M1 macrophages, IDR-1018 drives macrophages toward an intermediate M1-M2 phenotype, and Cathelicidin-WA promotes M2-like polarization [45,46,47]. In line with this immunomodulatory capacity of AMPs, our results showed that AP10W reduced the expression of M1-associated markers (CD86 and iNOS) and pro-inflammatory mediators (IL-1β, IL-6, and TNF-α), while increasing the expression of M2-associated markers (CD206 and Arg-1). These findings indicate that AP10W helps re-establish a pro-reparative immune microenvironment, which is likely to contribute substantially to its wound-healing activity.

Importantly, inflammatory resolution is closely coupled to other major reparative events, including re-epithelialization, angiogenesis, and matrix remodeling [1,13]. Timely control of inflammation is required for the transition from damage containment to tissue reconstruction, whereas adequate vascularization supplies oxygen and nutrients to support the proliferation of keratinocytes and fibroblasts. Consistent with this integrated view of wound healing, AP10W also exhibited marked pro-angiogenic activity. Specially, AP10W enhanced endothelial cell proliferation, migration, and tube formation in vitro, and these effects were paralleled in vivo by improved blood perfusion. Moreover, the increased expression of CD31 and α-SMA in AP10W-treated wounds suggests enhanced neovascularization together with improved vascular maturation. AP10W also significantly upregulated the expression of *VEGF*, *PDGF*, and *FGF* in HUVECs, which may at least partly account for its pro-angiogenic effects. Rather than acting on a single pathological event, AP10W therefore appears to improve the wound microenvironment in a coordinated manner by simultaneously modulating inflammation, vascular regeneration, and tissue remodeling. In this regard, AP10W resembles other multifunctional AMPs, such as hBDs and AMP-IBP5, which have also been reported to exert antimicrobial, immunomodulatory, pro-angiogenic, and tissue-reparative activities [18]. Such a mode of action may be particularly advantageous in chronic wounds, where multiple pathological defects coexist and mutually reinforce one another. However, this potential therapeutic relevance still requires further confirmation, as the present study evaluated AP10W only in an acute wound model. We have not yet assessed its efficacy under impaired-healing conditions, such as diabetic, ischemic, or infected wounds [48,49]. Although AP10W appears to target several pathological features shared by both acute and chronic wounds, the microenvironment of chronic wounds is far more complex and is often accompanied by persistent oxidative stress and dysregulated immune responses [43]. Whether AP10W can exert therapeutic effects in chronic wounds remains to be determined. Therefore, we will further validate its therapeutic potential in disease-relevant models of chronic or hard-to-heal wounds.

**Figure 6:**
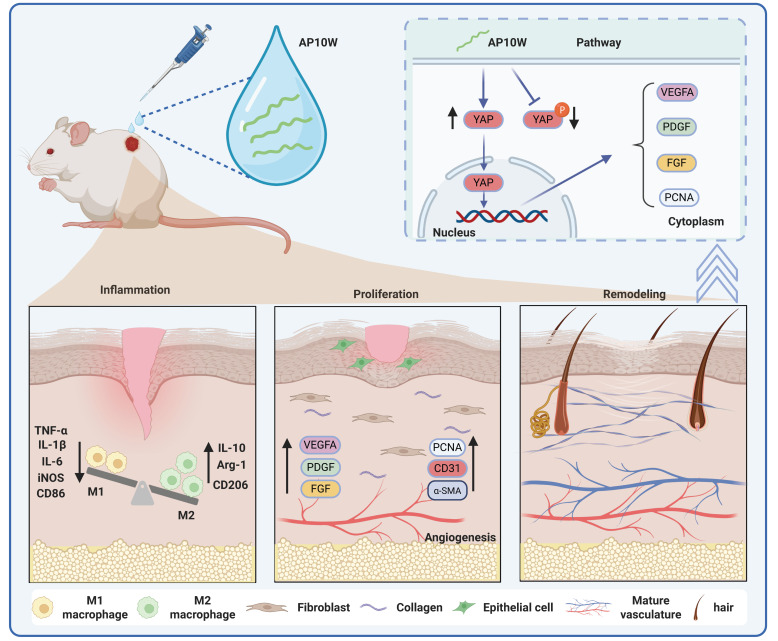
A schematic representation illustrates that AP10W effectively accelerates cutaneous repair by promoting keratinocyte, fibroblast, and endothelial cell proliferation and migration, enhancing angiogenesis, facilitating macrophage polarization toward a pro-healing phenotype, and improving matrix remodeling through activation of YAP signaling. Solid arrows represent mechanisms verified by our inhibitor/Western blotting experiments. The schematic figure was created by the authors using BioRender (https://www.biorender.com/; accessed on 9 April 2026).

Mechanistically, one of the most notable findings of the present study is the implication of YAP signaling in AP10W-mediated wound repair. YAP is a core downstream transcriptional co-activator of Hippo pathway, an evolutionarily conserved signaling cascade involved in organ development, epithelial homeostasis, tissue regeneration, wound healing and immune modulation [19,20]. When the Hippo pathway is active, YAP is phosphorylated by LATS1/2 and retained in the cytoplasm; when the pathway is inactive, dephosphorylated YAP translocates into the nucleus and interacts with transcription factors to regulate target gene expression [50]. YAP has been shown to be essential for wound healing, as YAP deficiency markedly delays wound closure [19]. Conversely, pharmacological activation of YAP promotes regenerative cutaneous repair in pigs and human models and enhances keratinocyte proliferation and wound closure capacity in vitro [22]. In keratinocytes and fibroblasts, YAP activation drives proliferation and migration required for re-epithelialization and dermal reconstruction [23]. In our study, AP10W increased YAP expression and promoted its nuclear translocation, whereas pharmacological inhibition of YAP largely abolished AP10W-induced proliferation, migration, and repair-related gene expression in fibroblasts and keratinocytes. Moreover, YAP inhibition also markedly impaired AP10W-induced tube formation in endothelial cells, consistent with previous reports that extracellular vesicle-like particles can promote angiogenesis and wound healing through YAP activation [51]. Collectively, these findings strongly suggest that YAP activation is functionally required, at least in part, for the pro-healing actions of AP10W (Figure 6). Nevertheless, although we identified YAP as a key mediator of AP10W activity [19,22], the present mechanistic investigation remains preliminary. In particular, the upstream activators and downstream transcriptional targets of YAP were not fully characterized [50]. Future studies will systematically investigate the upstream regulators that may couple AP10W to YAP activation, including Hippo pathway components, receptor signaling, and cytoskeletal dynamics, as well as the downstream effectors through which YAP mediates AP10W-induced repair responses [20,50]. In addition, other signaling pathways involved in wound healing, such as MAPK, PI3K/Akt, and STAT3, may also contribute to the biological effects of AP10W [27,31].

## 5. Conclusions

In conclusion, AP10W is a promising multifunctional antimicrobial peptide with significant potential for cutaneous wound repair. In addition to its known antimicrobial activity, AP10W directly promoted the proliferation and migration of keratinocytes, fibroblasts, and endothelial cells, enhanced angiogenesis and vascular maturation, and improved blood perfusion in vivo. It also modulated the inflammatory microenvironment by suppressing pro-inflammatory mediators and promoting macrophage polarization toward a pro-reparative M2 phenotype. Consistently, histological analyses showed that AP10W reduced inflammatory cell infiltration, enhanced collagen deposition and organization, and facilitated skin appendage regeneration, ultimately leading to faster and higher-quality wound healing.

Mechanistically, the pro-healing effects of AP10W were mediated, at least in part, through activation of YAP signaling. AP10W increased YAP expression and nuclear translocation, whereas pharmacological inhibition of YAP markedly attenuated its effects on cell proliferation, migration, and angiogenesis. Collectively, these findings expand the biological role of AP10W from an antimicrobial molecule to an active regulator of tissue repair and support its potential as a therapeutic candidate for complex or chronic wounds.

## Figures and Tables

**Table 1:** Primer sequences for qRT-PCR in human.

Gene	Forward (5′-3′)	Reverse (5′-3′)
*VEGFA*	GAGGGCAGAATCATCACGAA	GGTCTCGATTGGATGGCAGTA
*PDGF*	AGCGCCCATTTTTCATTCCCTA	GGTTTTCTCTTTGCAGCGAGGC
*FGF2*	GCAGGAGAGAGGAAGCCTTG	CGTGAGAGCAGAGCATGTGA
*GAPDH*	GTCTCCTCTGACTTCAACAGCG	ACCACCCTGTTGCTGTAGCCAA

**Table 2:** Primer sequences for qRT-PCR in mouse.

Gene	Forward (5′-3′)	Reverse (5′-3′)
*Col1a1*	CGCCATCAAGGTCTACTGC	GAATCCATCGGTCATGCTCT
*Col3a1*	CCCACAGCCTTCTACACCT	ACCCATTCCTCCCACTCC
*Ccnd1*	AAAATGCCAGAGGCGGATGA	GAGGGGGTCCTTGTTTAGCC
*Acta2*	CGGCCAAACCCTGTGAAGGA	GCCCCTGGGGCTCTTGTGGT
*Il-1β*	TTCAGGCAGGCAGTATCACTC	GAAGGTCCACGGGAAAGACAC
*Il-6*	TAGTCCTTCCTACCCAAATTTCC	TTGGTCCTTAGCCACTCCTTC
*T* *nf* *-* *α*	ATCCTGTCCAAACTAAGGCTCG	ACCTCTTTAGCATAGTAGTCCGC
*i* *NOS*	GAGACAGGGAAGTCTGAAGCAC	CCAGCAGTAGTTGCTCCTCTTC
*Il-10*	GCCCTTTGCTATGGTGTC	TCTCCCTGGTTTCTCTTCC
*Arg* *-* *1*	TGGCTTGCGAGACGTAGAC	GCTCAGGTGAATCGGCCTTT
*Gapdh*	AGGTCGGTGTGAACGGATTT	TGTAGACCATGTAGTTGAGGTCA

## Data Availability

The original contributions presented in this study are included in the article/Supplementary Materials. Further inquiries can be directed to the corresponding authors.

## Supplementary Materials

The following supporting information can be downloaded at https://www.mdpi.com/article/10.3390/biom16050720/s1: Figure S1: Relative epithelial thickness at the wound site on day 14 (*n* = 3). Table S1: Comparing AP10W with other previously published proteins [24,25,52,53,54,55,56,57,58,59,60,61,62,63,64,65,66,67,68,69,70,71,72,73,74,75,76]. Western blot original images.
