# Supplementary material for: The Multifunctional Peptide AP10W Enhances Skin Wound Healing Through Macrophage Reprogramming and Angiogenesis
Source: Biomolecules. 2026 May 13;16(5):720. doi: 10.3390/biom16050720 (PMC13204542; doi:10.3390/biom16050720)

GAPDH

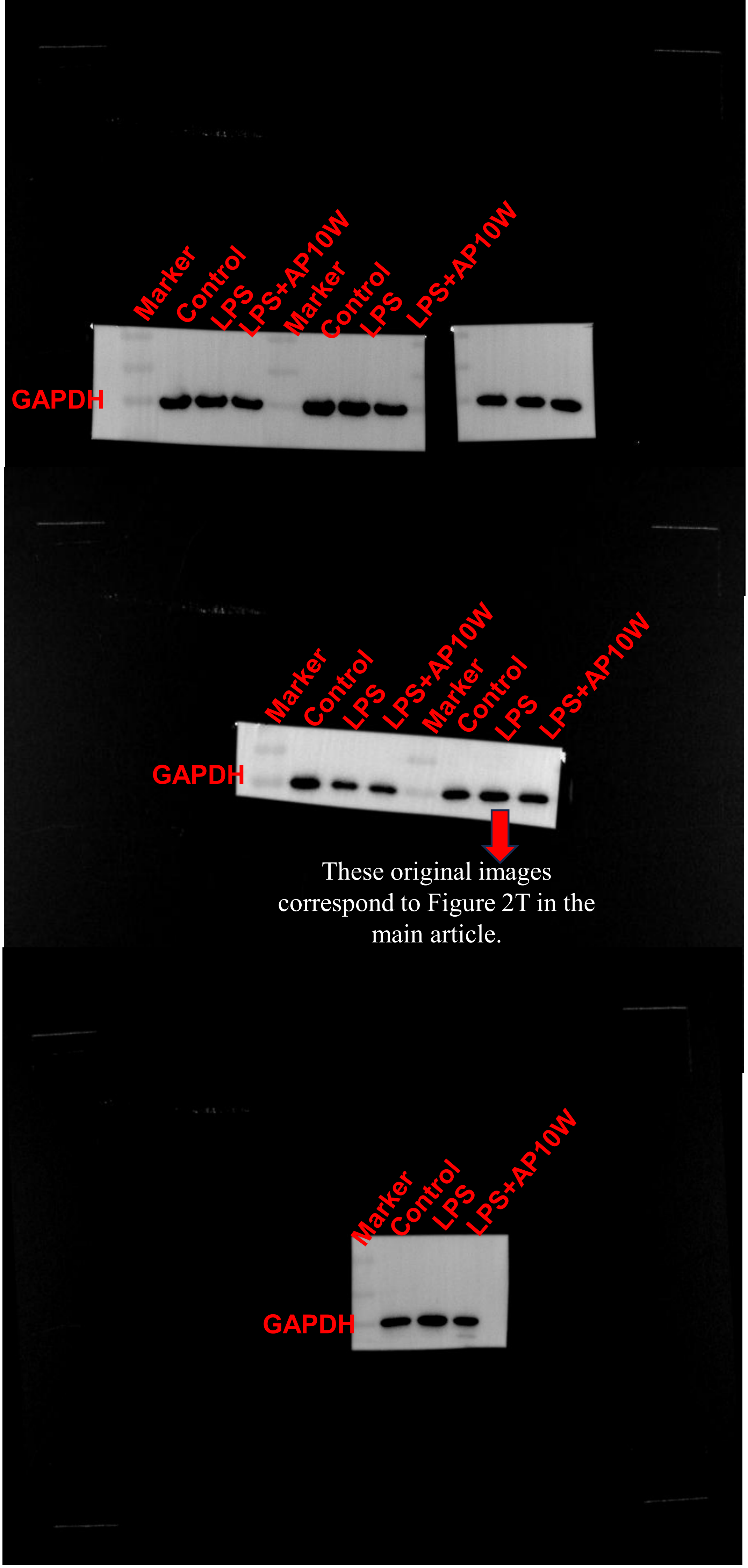

These original images correspond to Figure 2T in the main article.

CD86

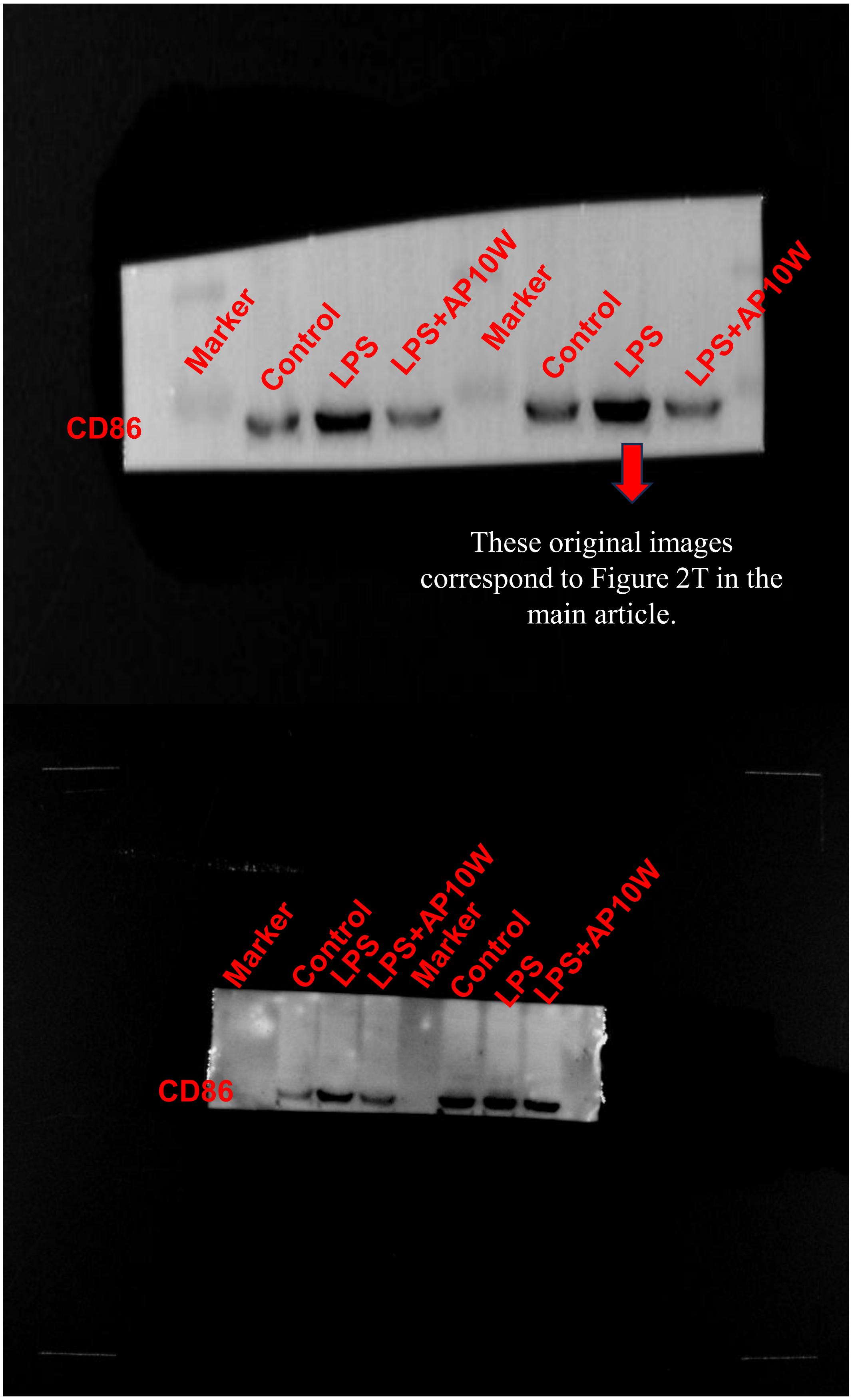

These original images correspond to Figure 2T in the main article.

CD206

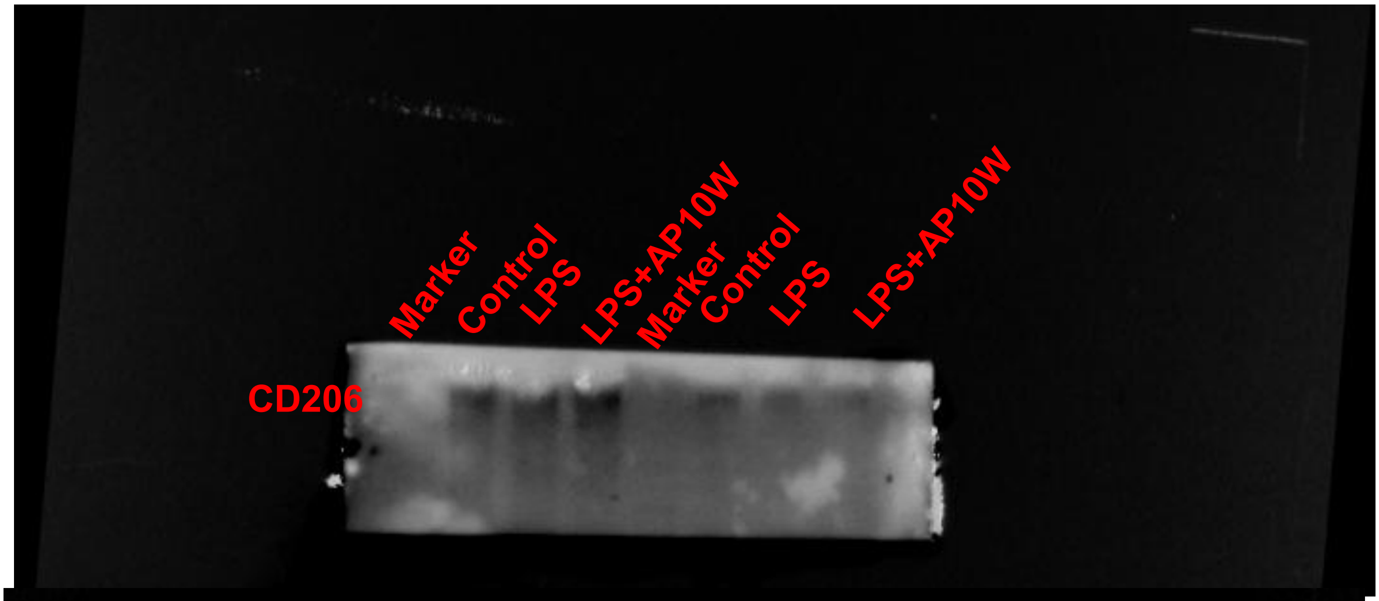

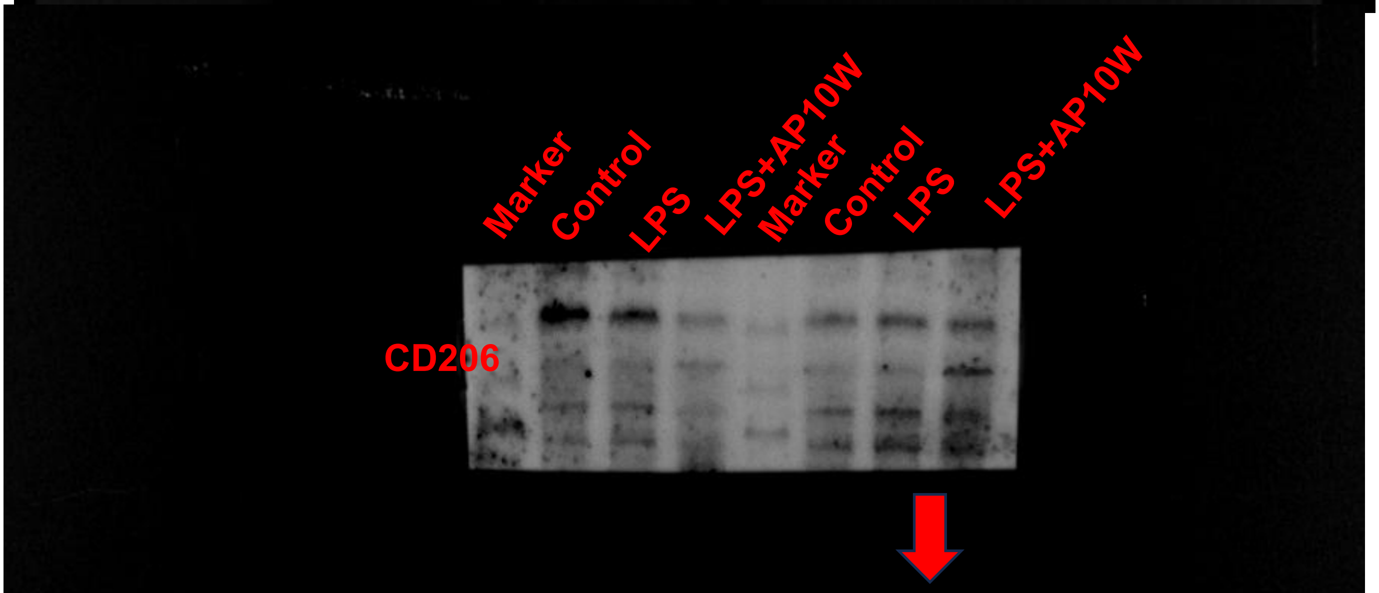

These original images correspond to Figure 2T in the main article.

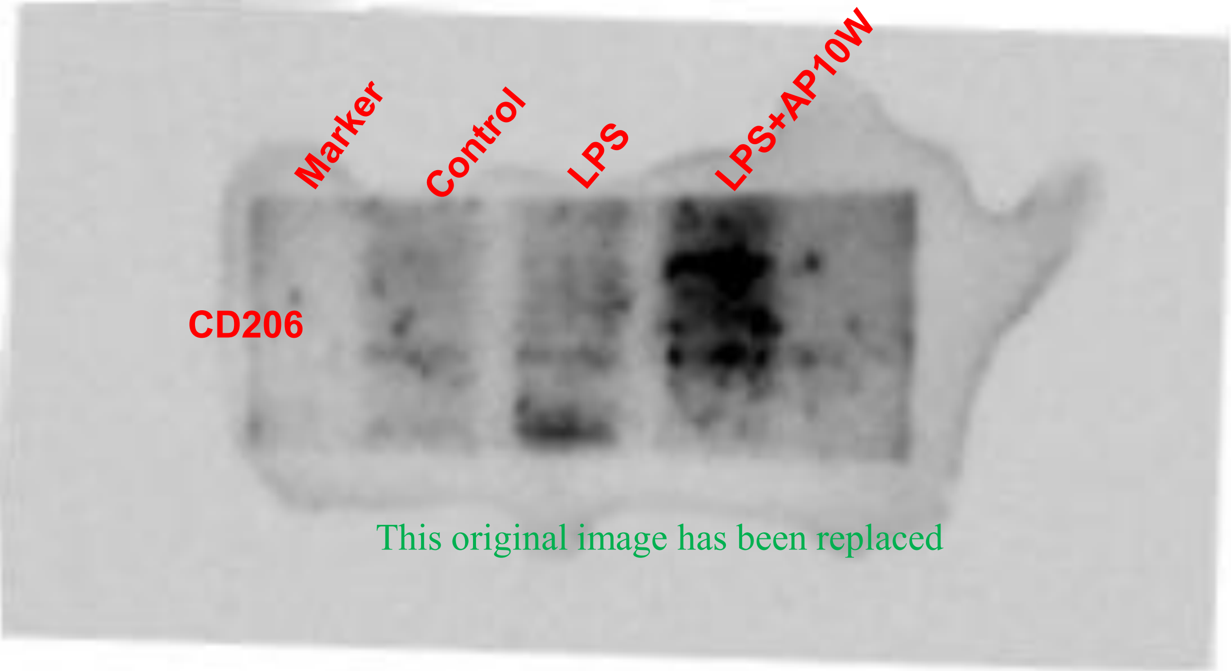

L929

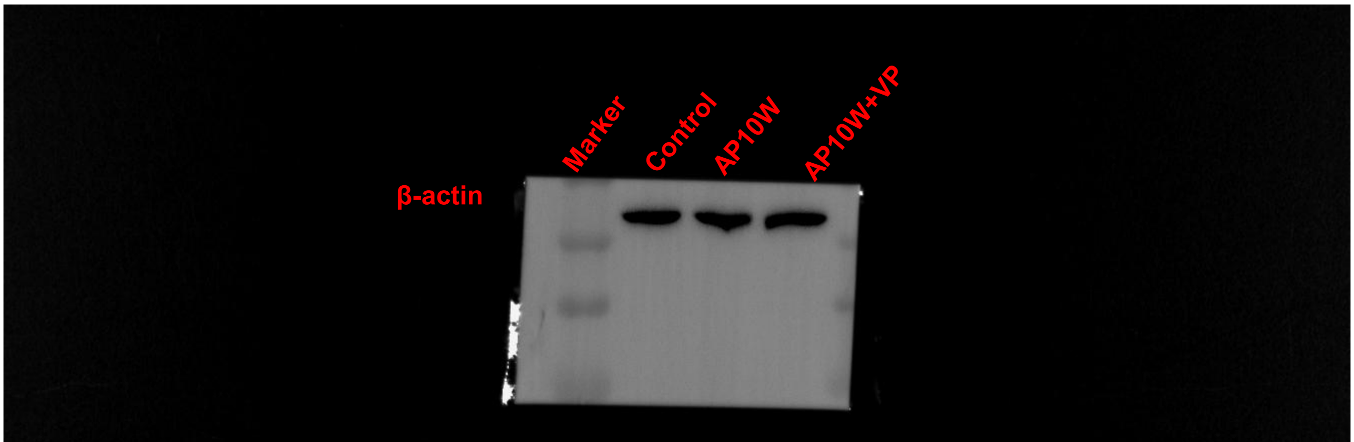

These original images correspond to Figure 5A in the main article.

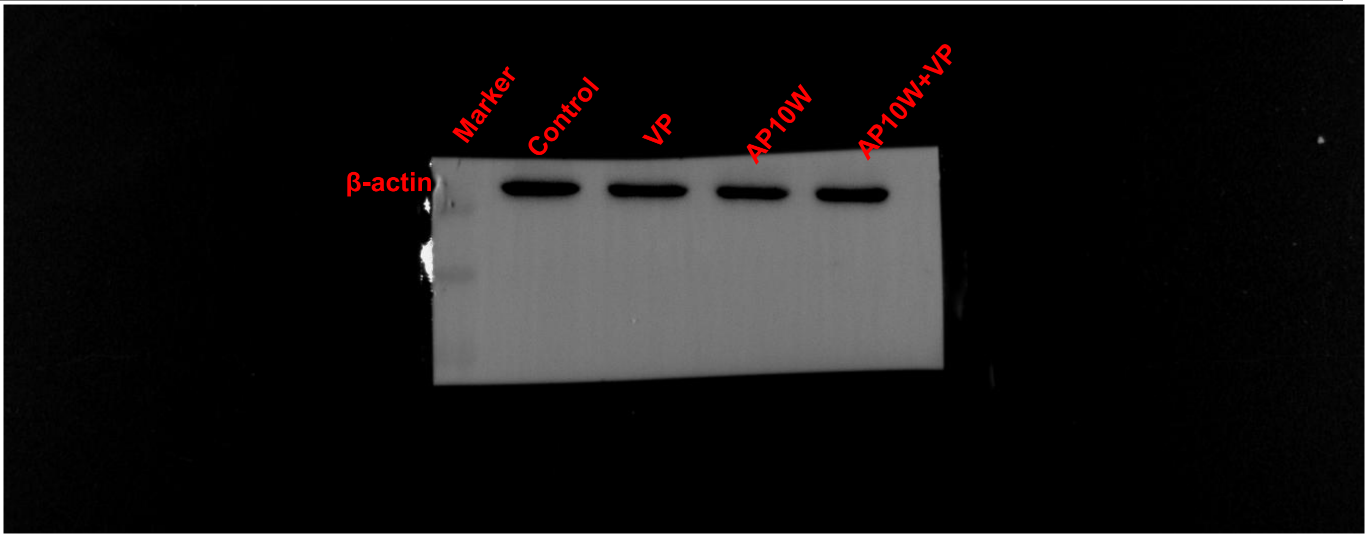

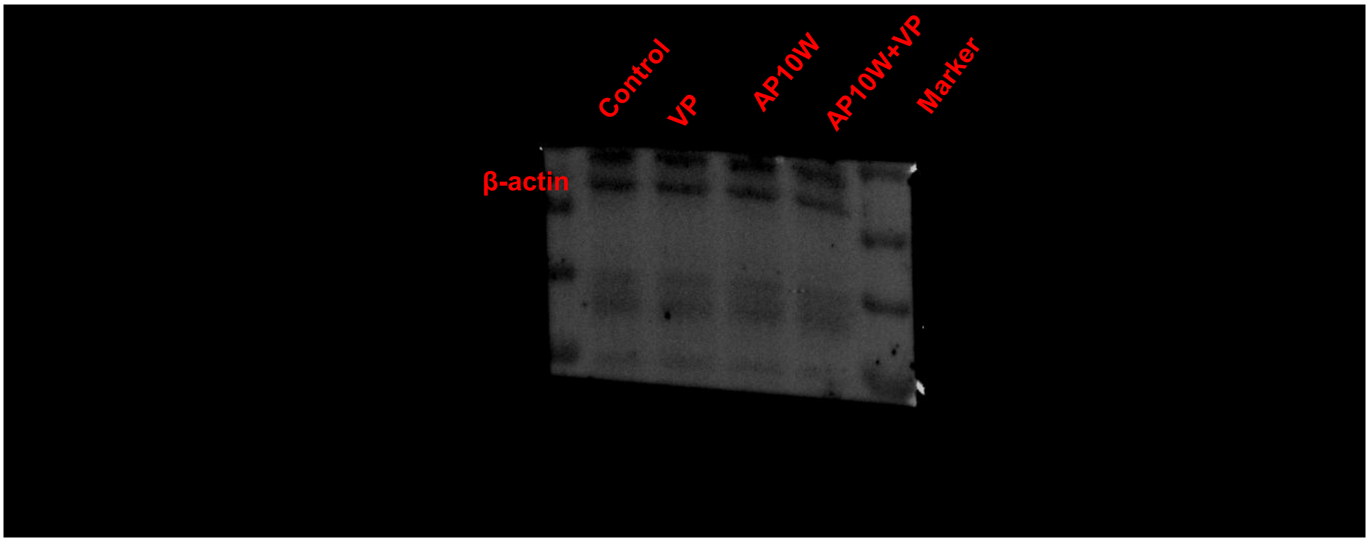

L929

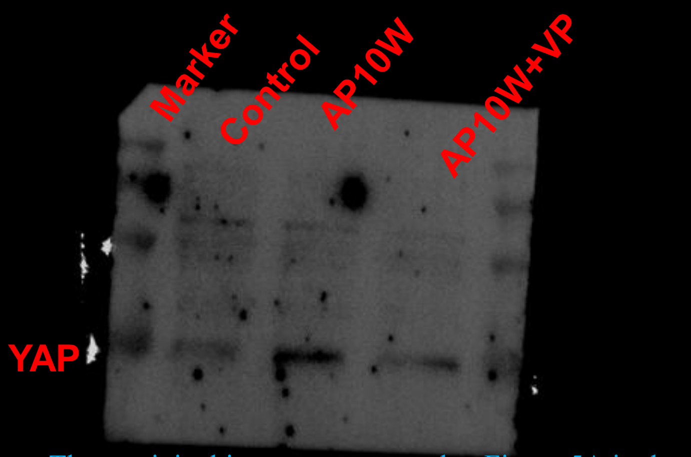

These original images correspond to Figure 5A in the main article.

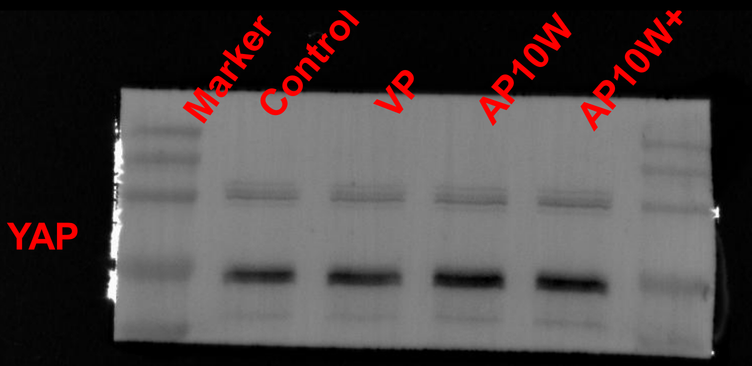

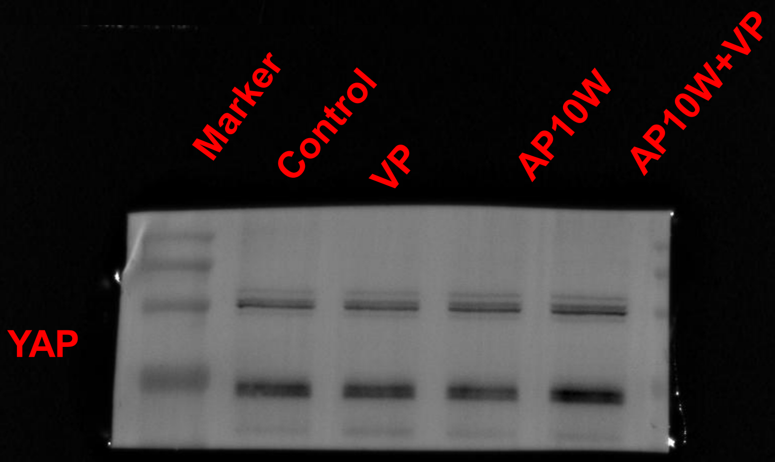

L929

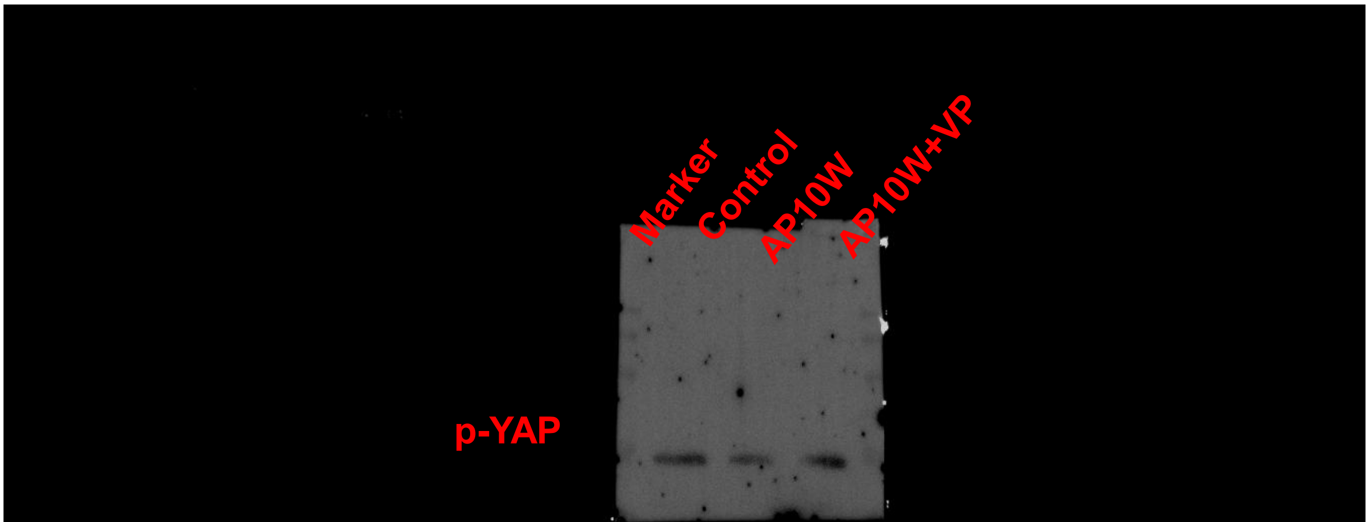

These original images correspond to Figure 5A in the main article.

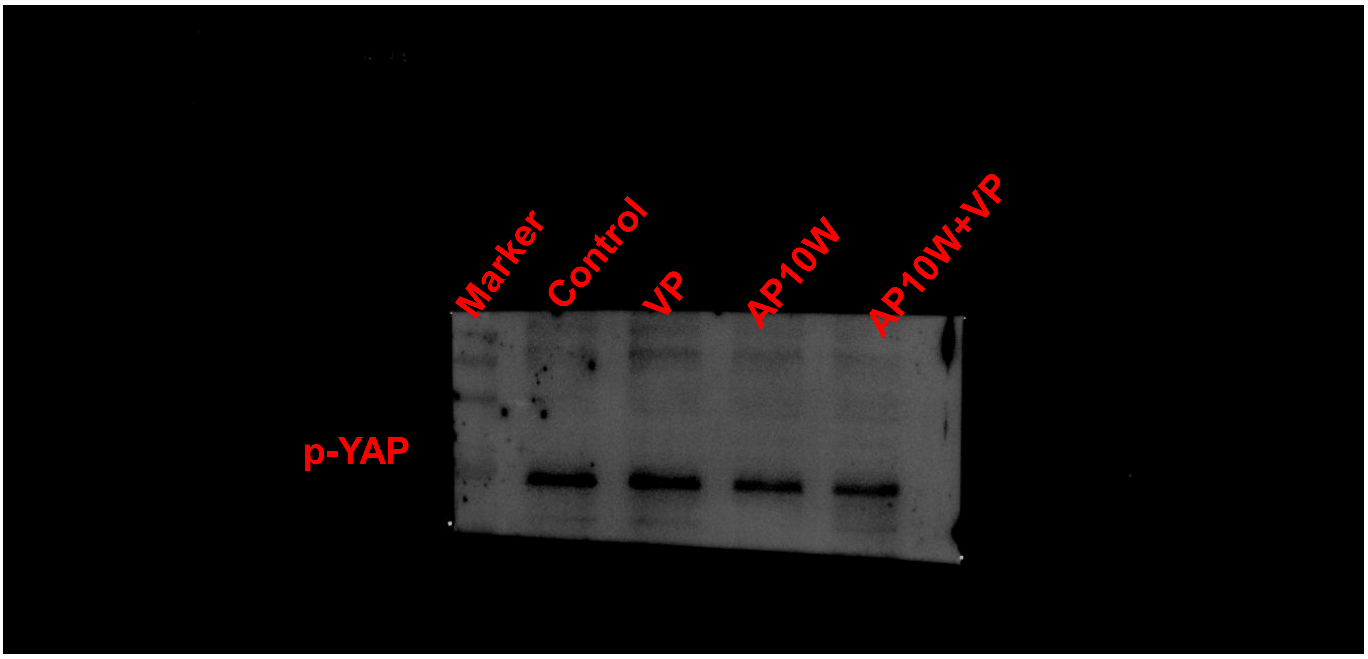

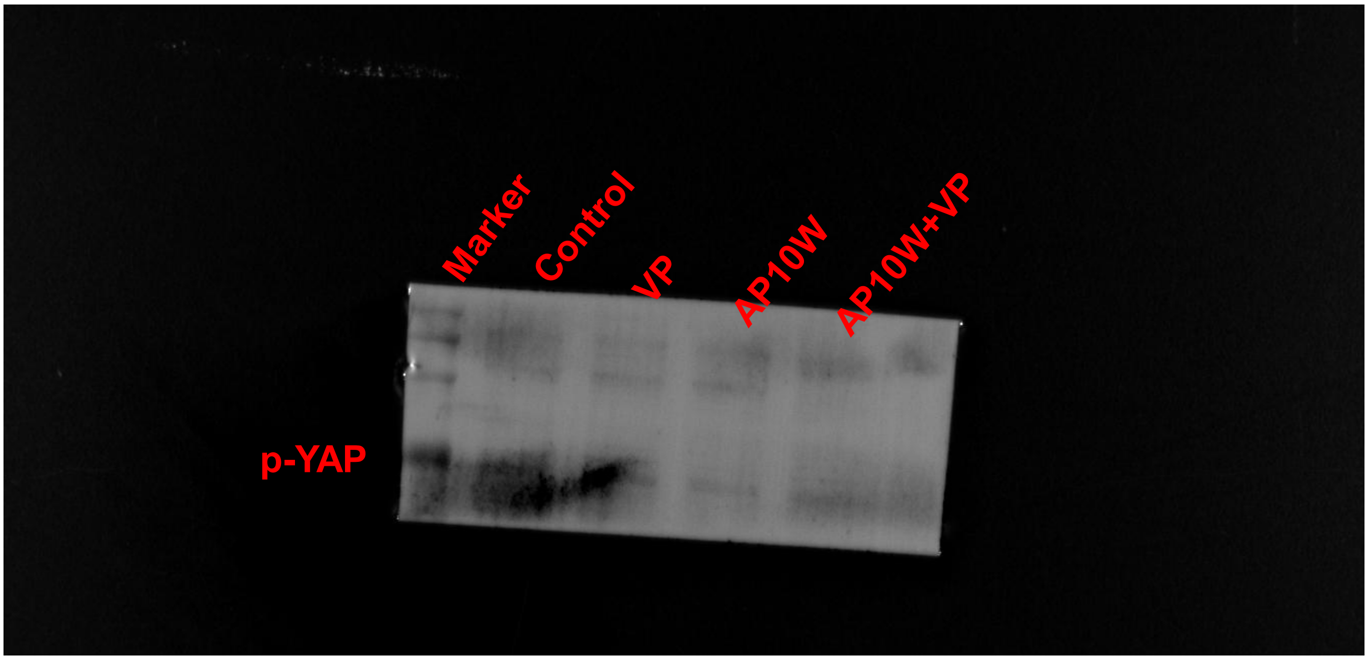

L929

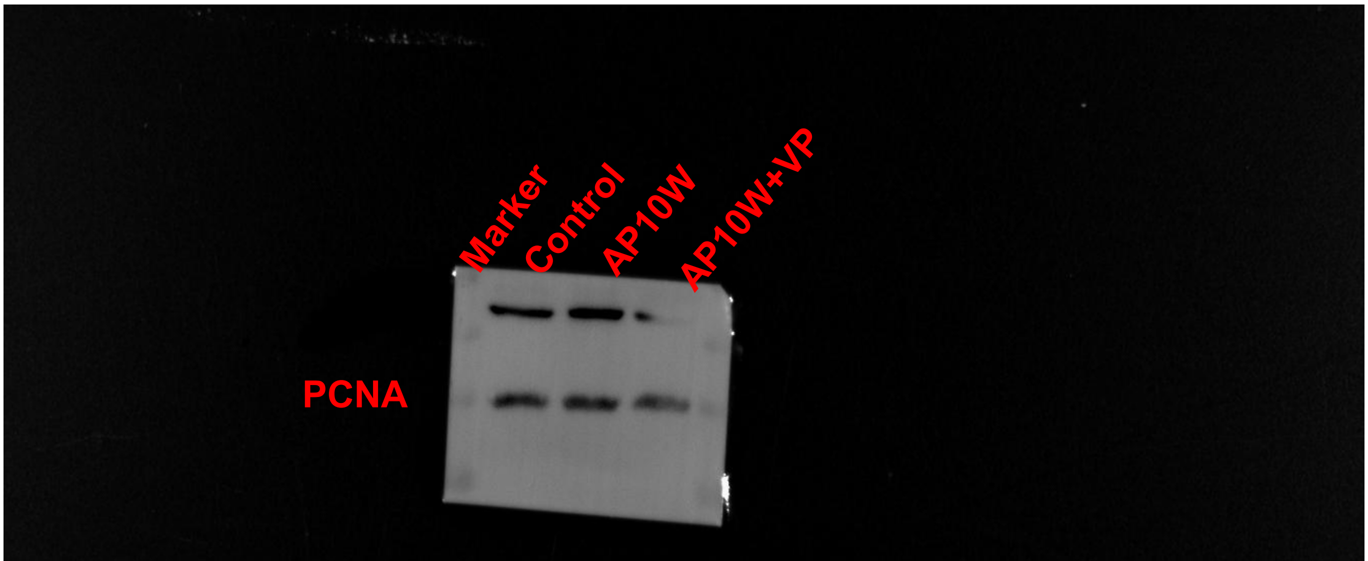

These original images correspond to Figure 5A in the main article.

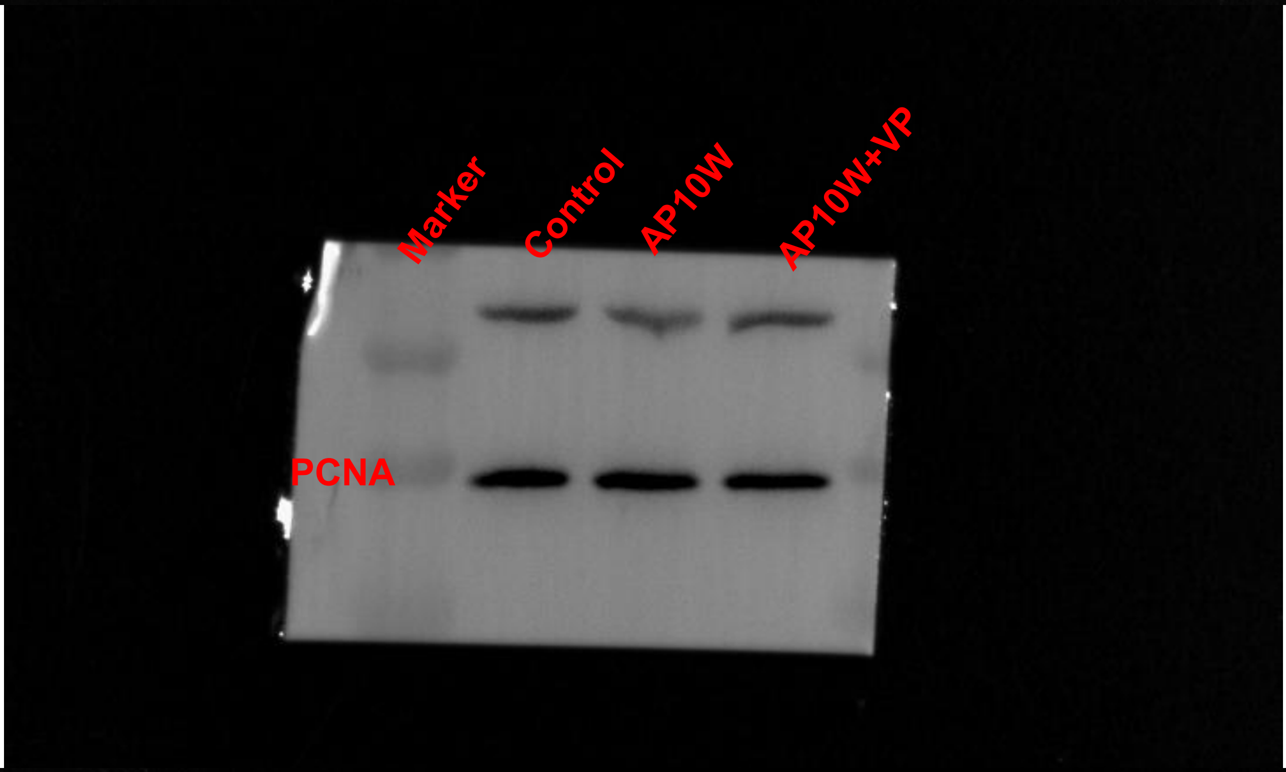

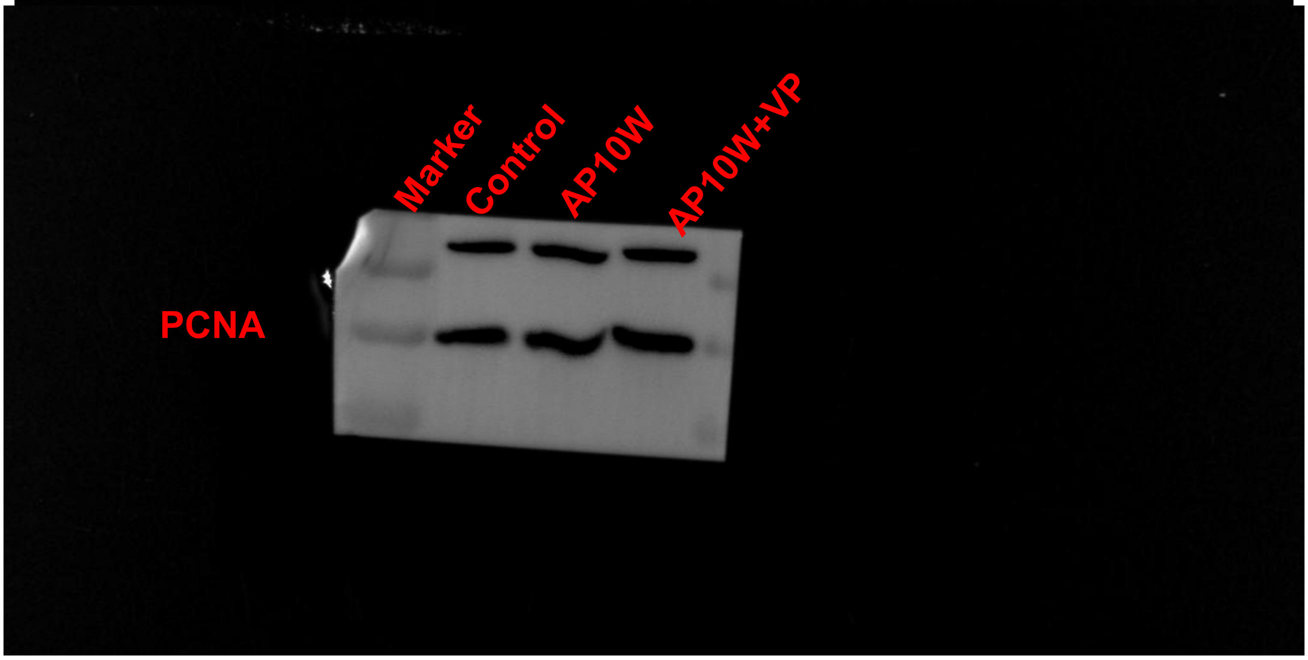

HaCaT

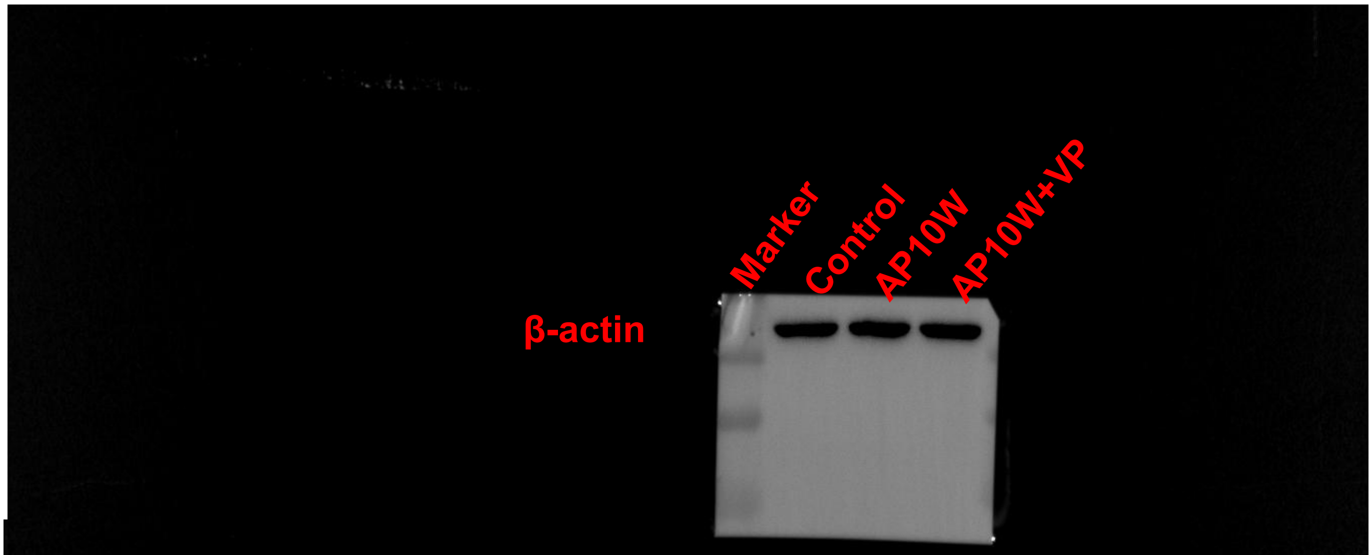

These original images correspond to Figure 5B in the main article.

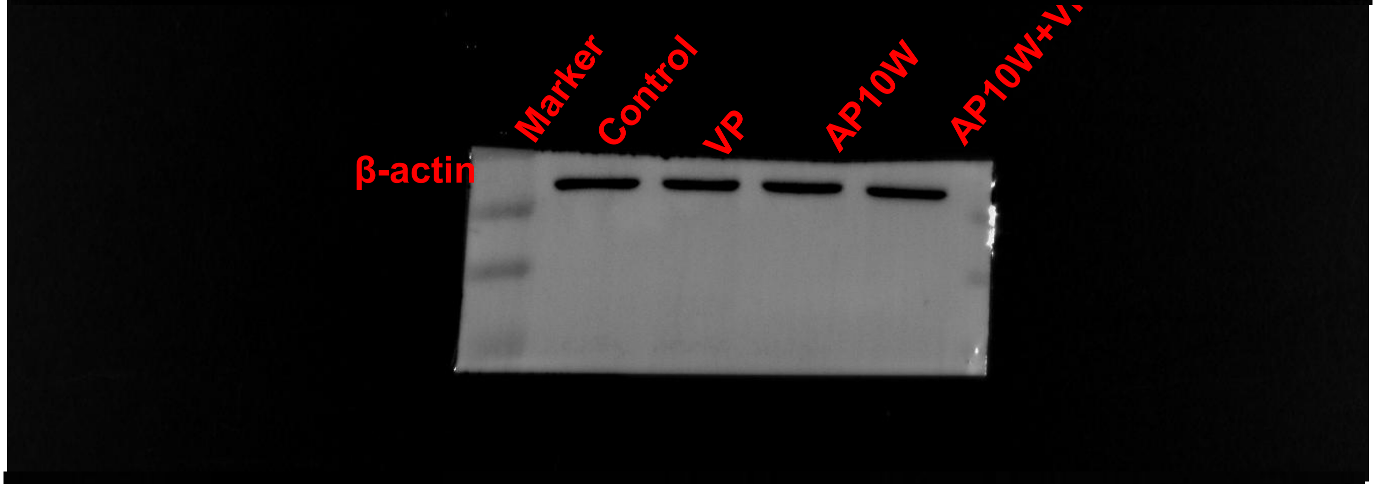

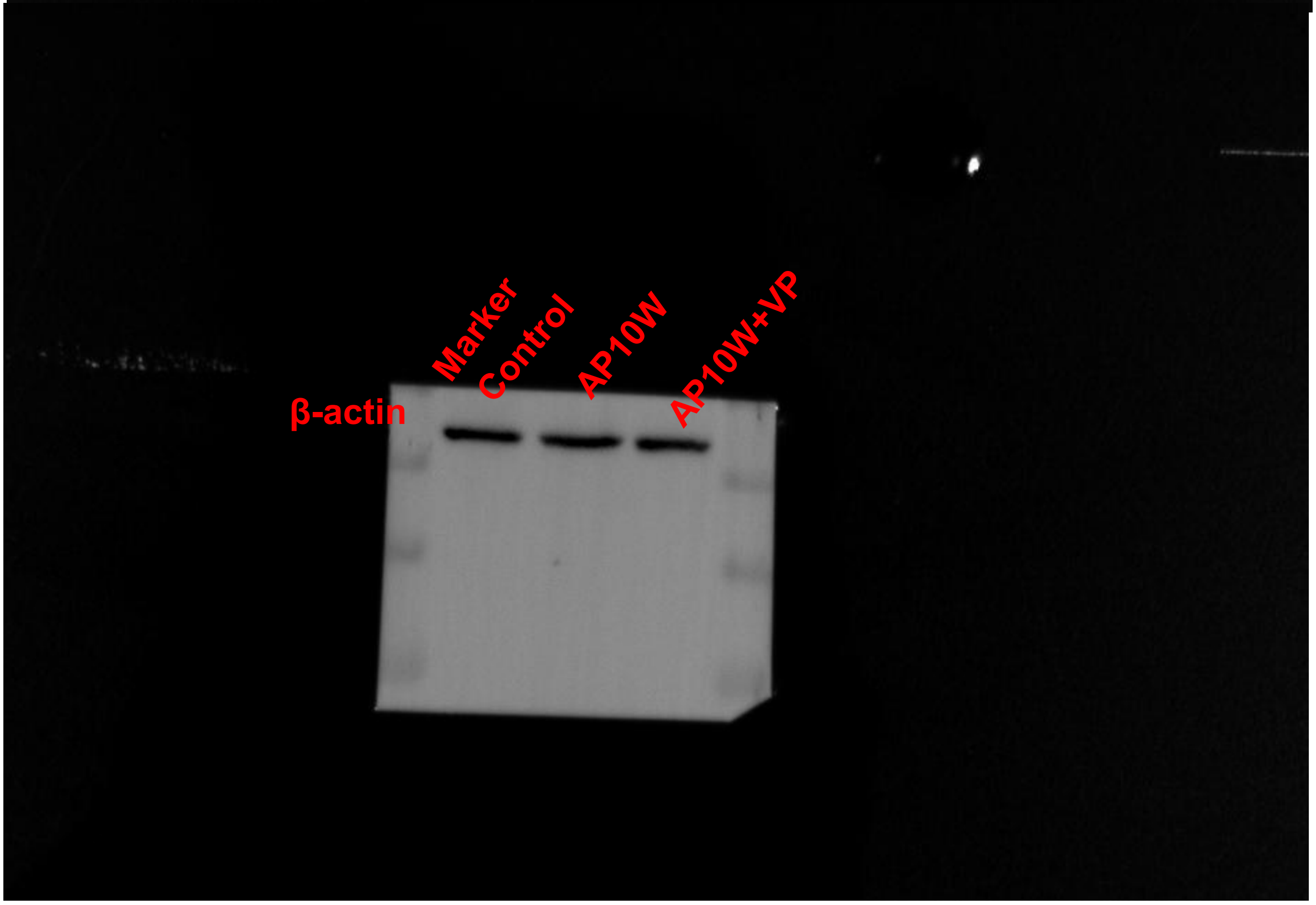

HaCaT

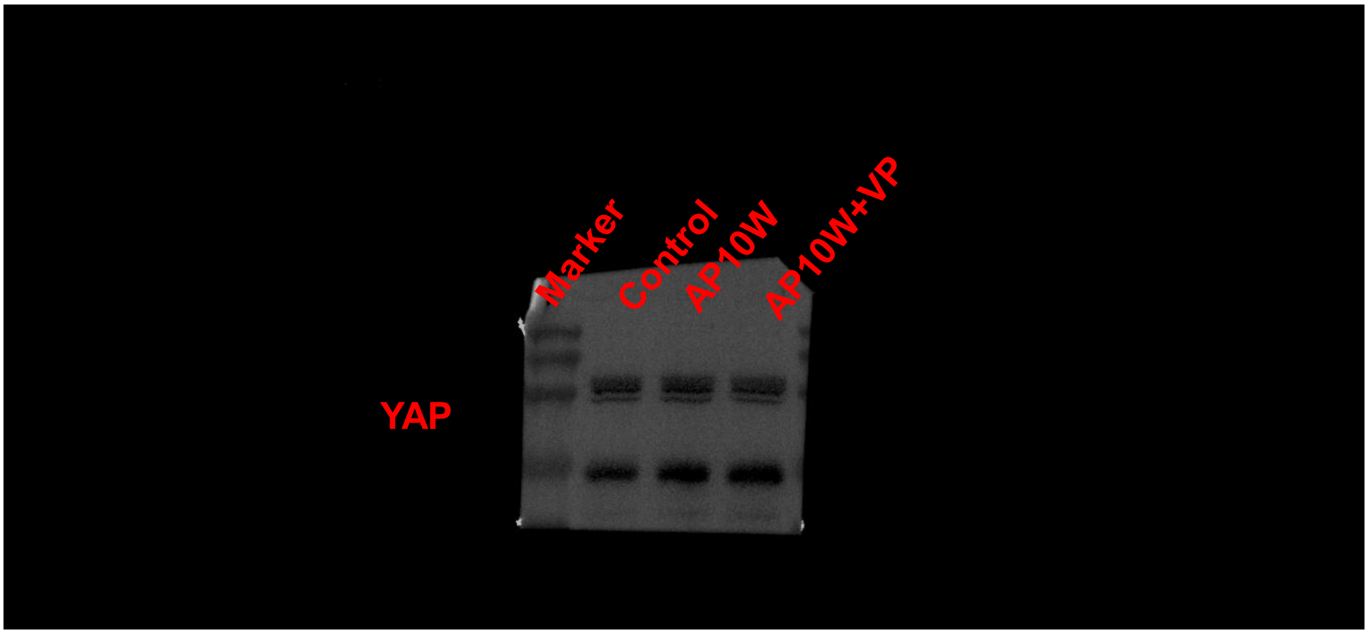

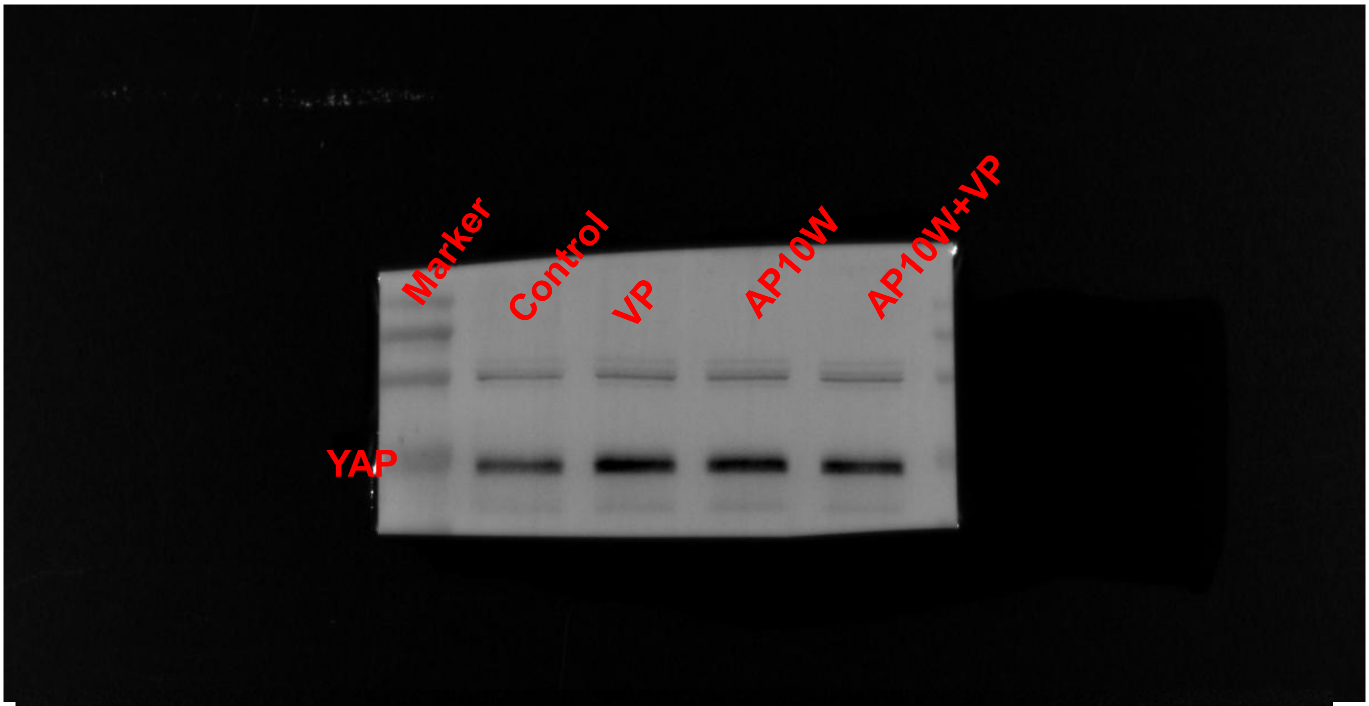

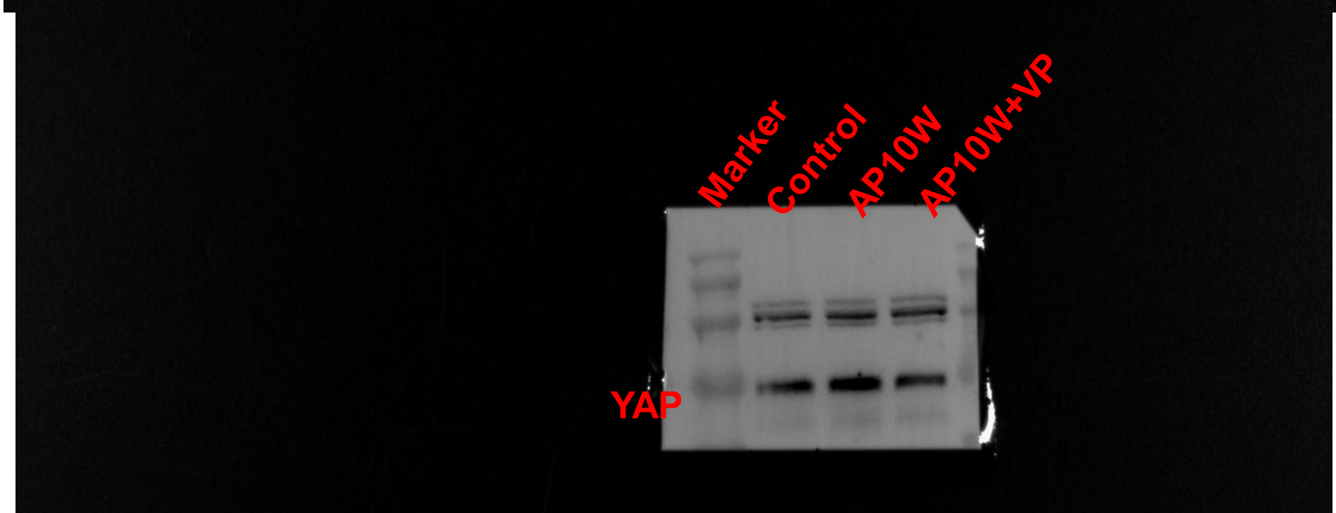

These original images correspond to Figure 5B in the main article.

HaCaT

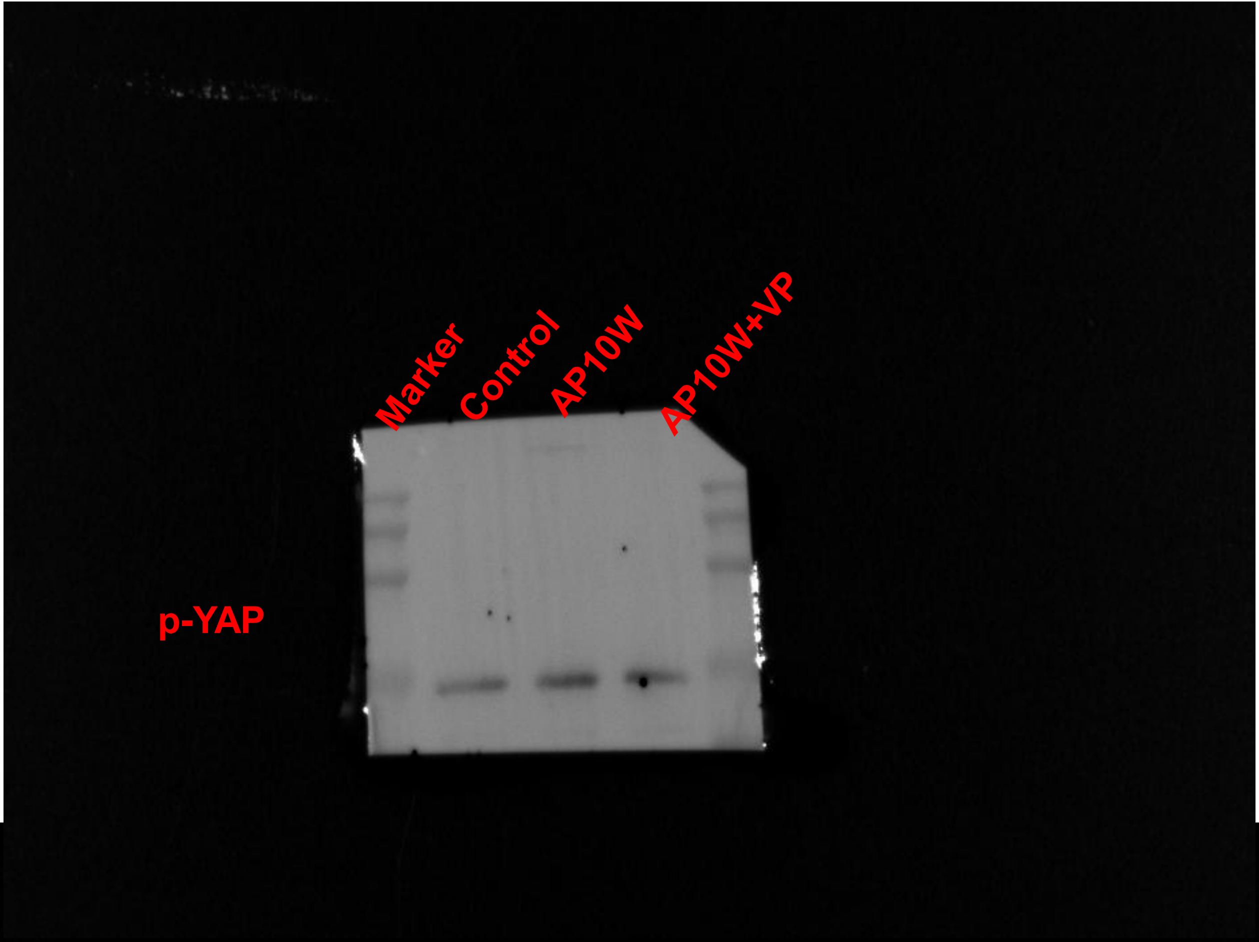

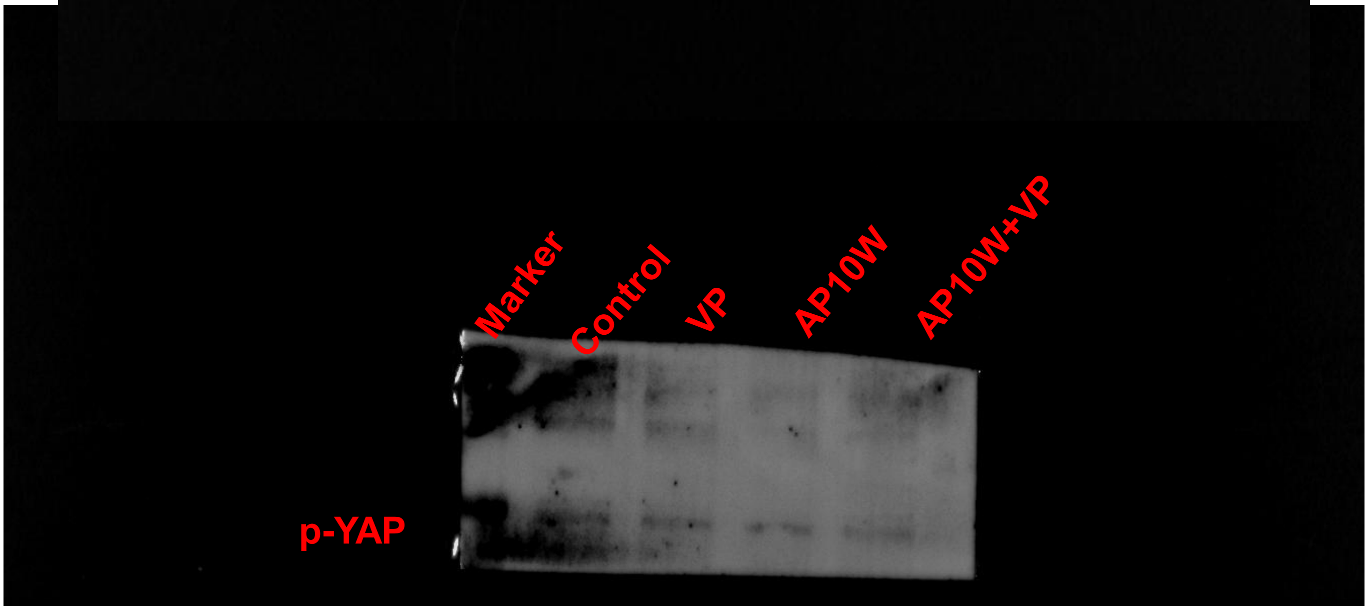

This original image has been replaced

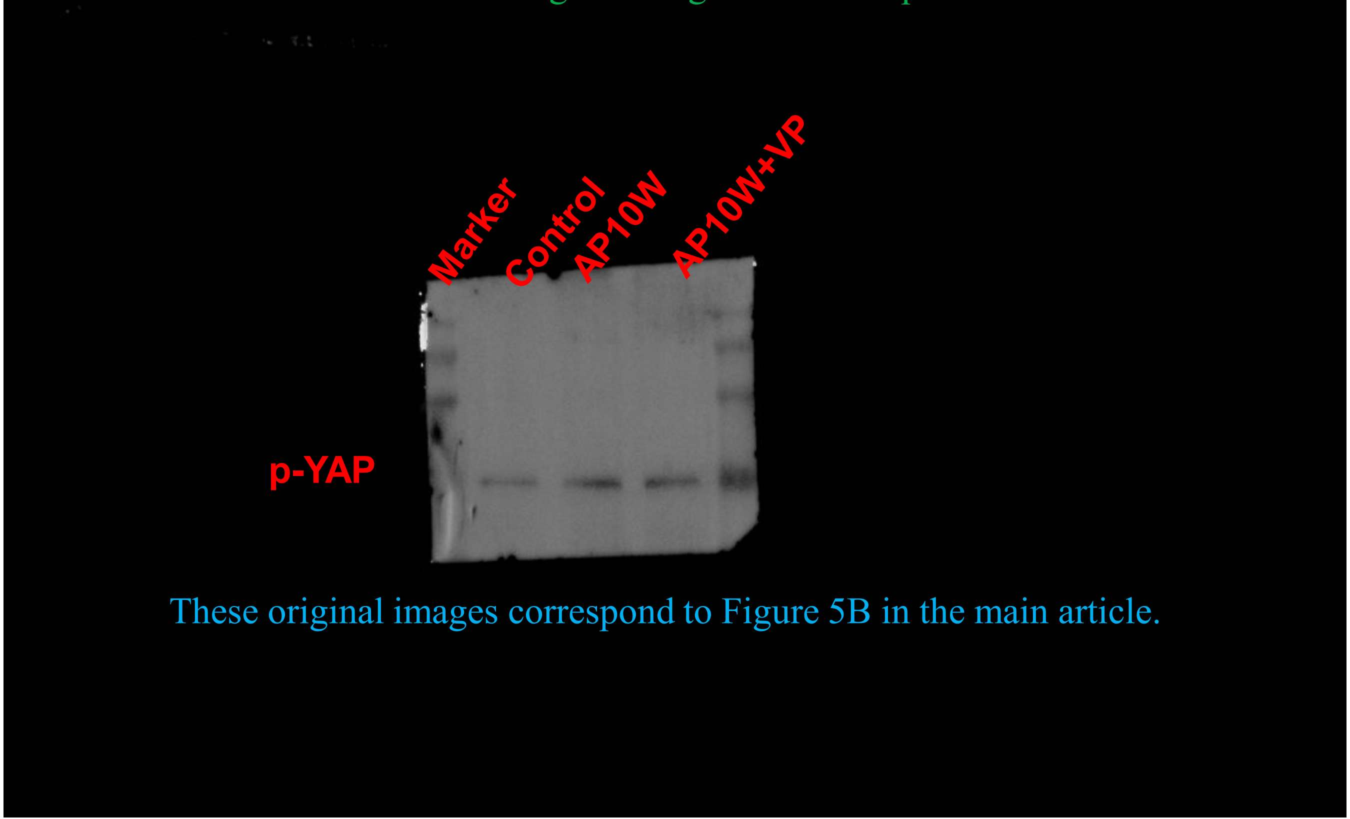

These original images correspond to Figure 5B in the main article.

HaCaT

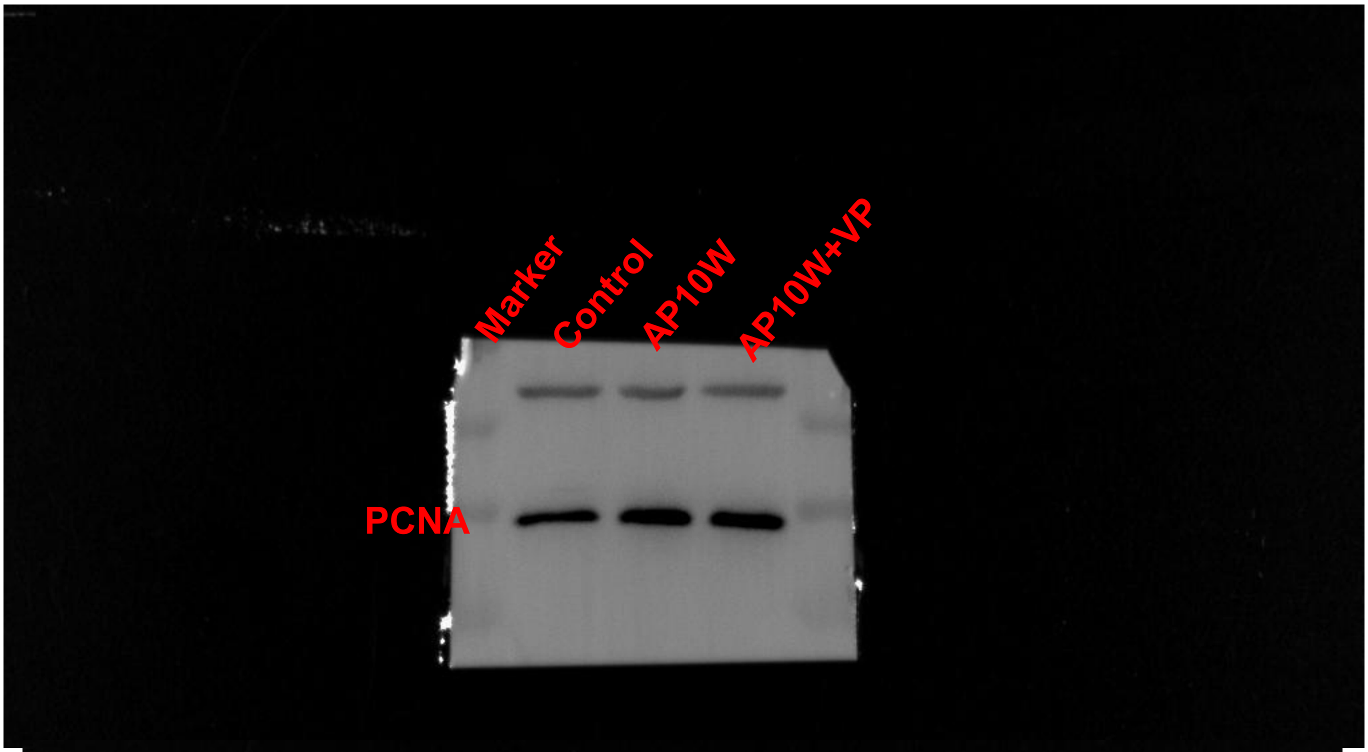

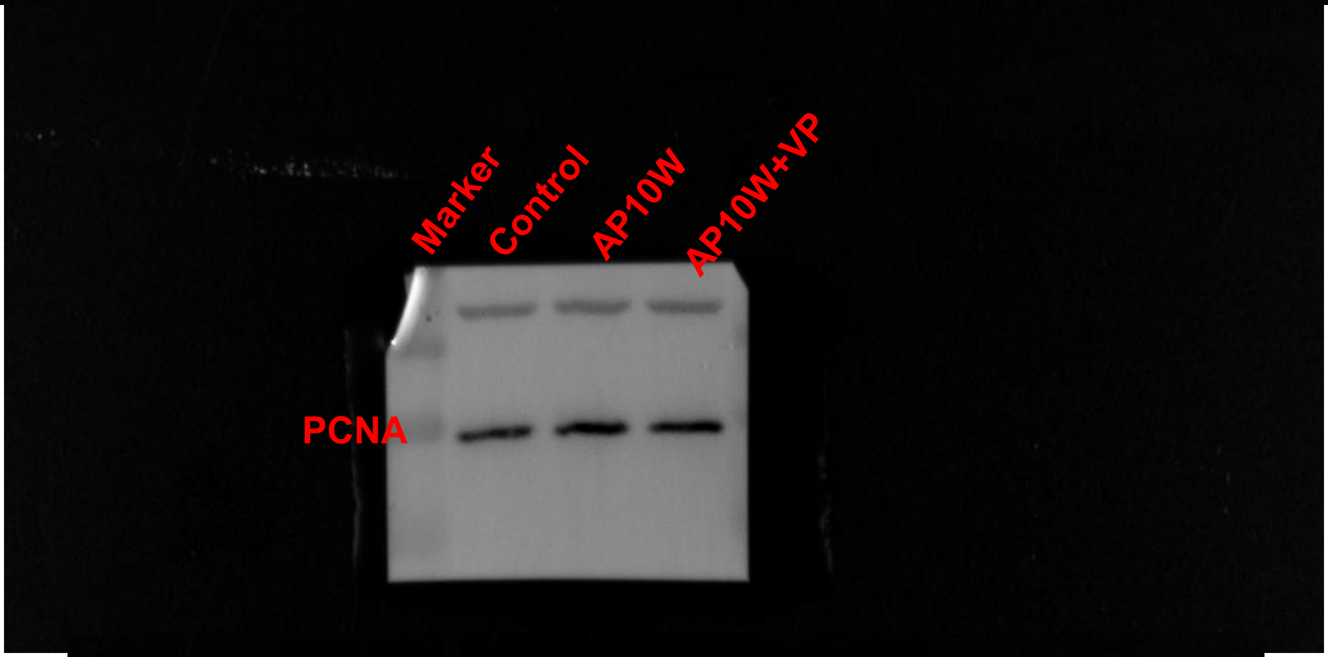

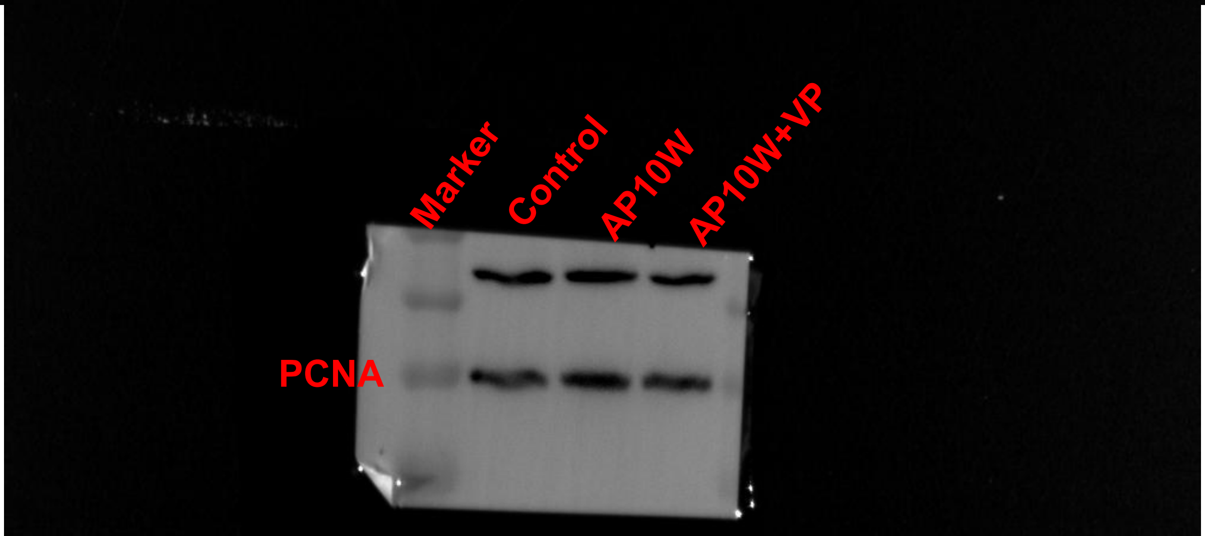

These original images correspond to Figure 5B in the main article.

HUVEC

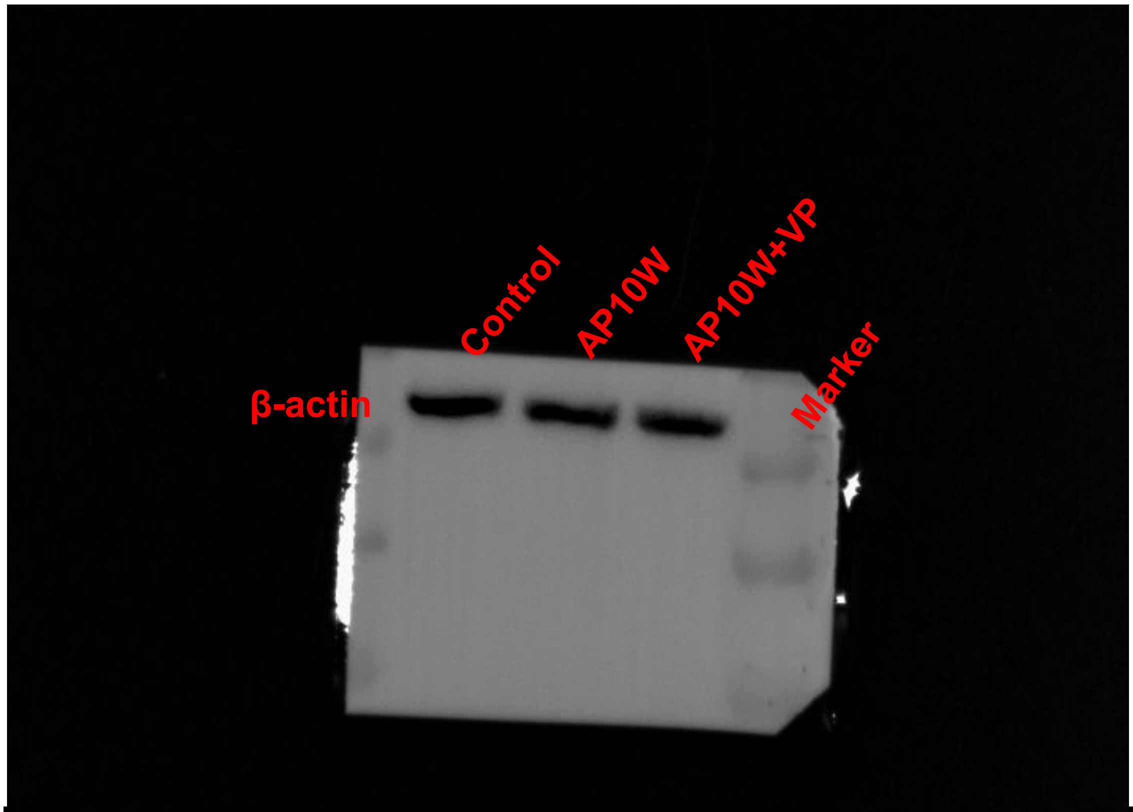

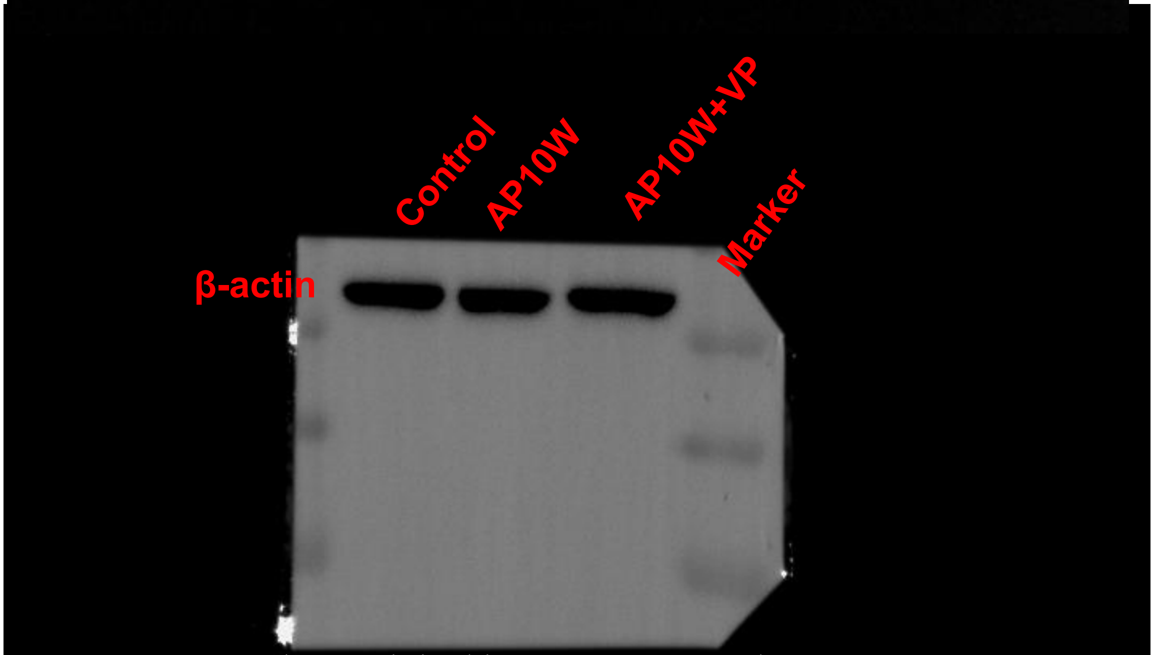

These original images correspond to Figure 5C in the main article.

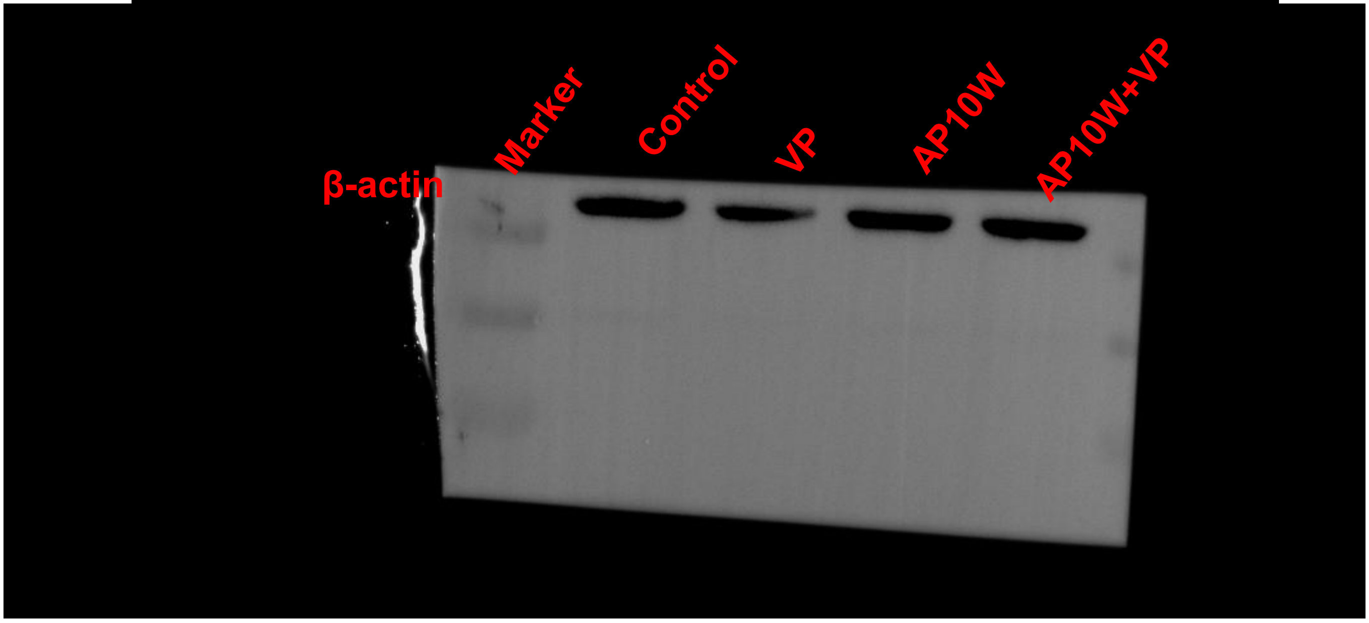

HUVEC

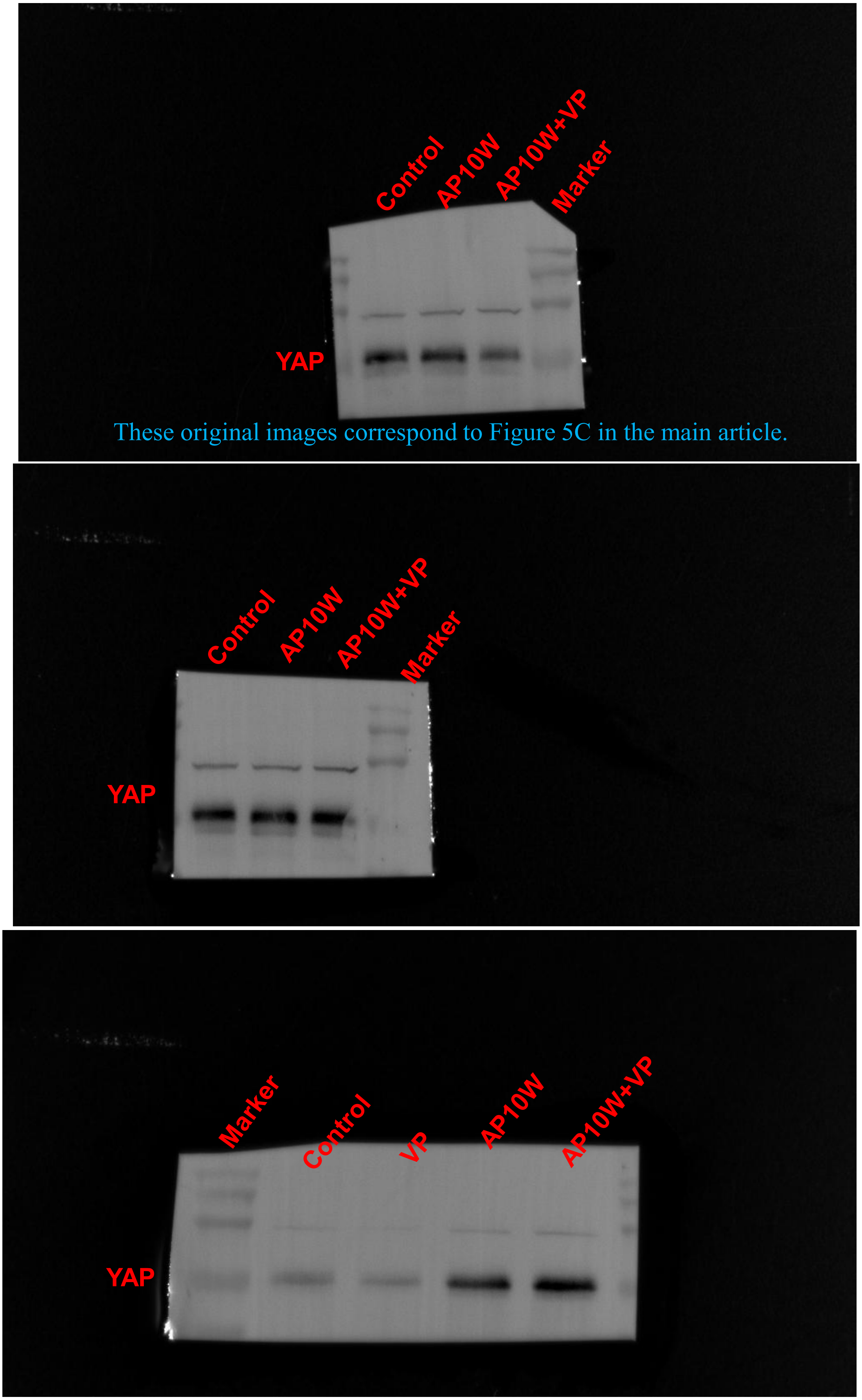

These original images correspond to Figure 5C in the main article.

HUVEC

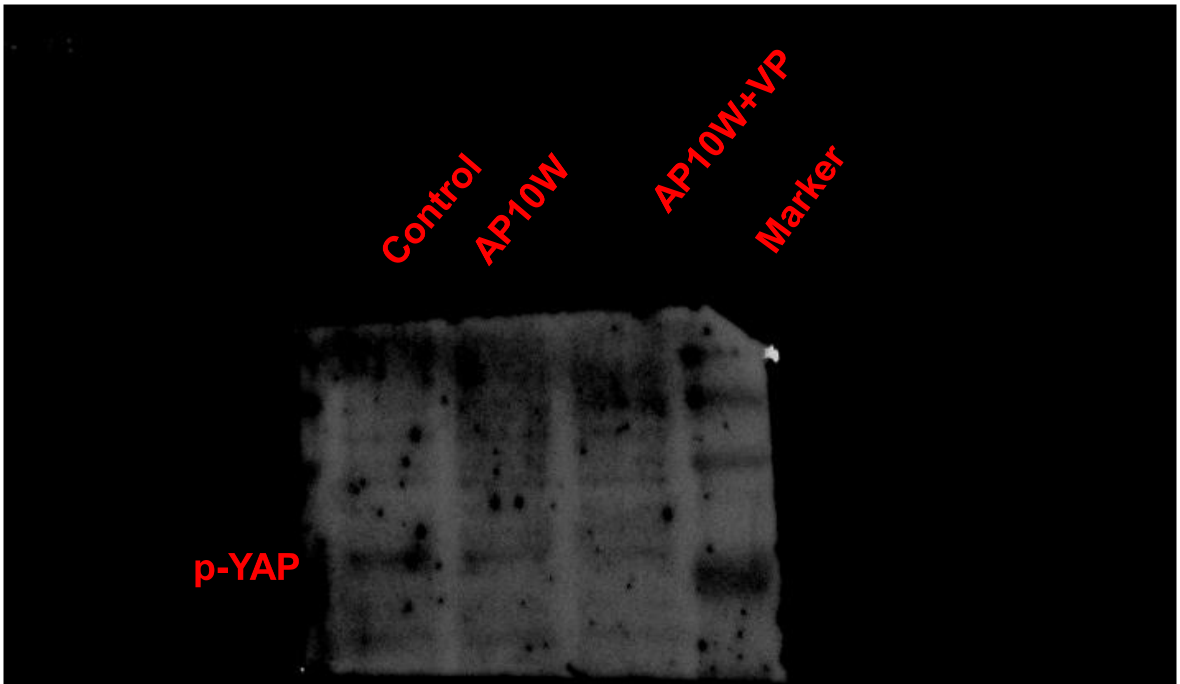

These original images correspond to Figure 5C in the main article.

This original image has been replaced

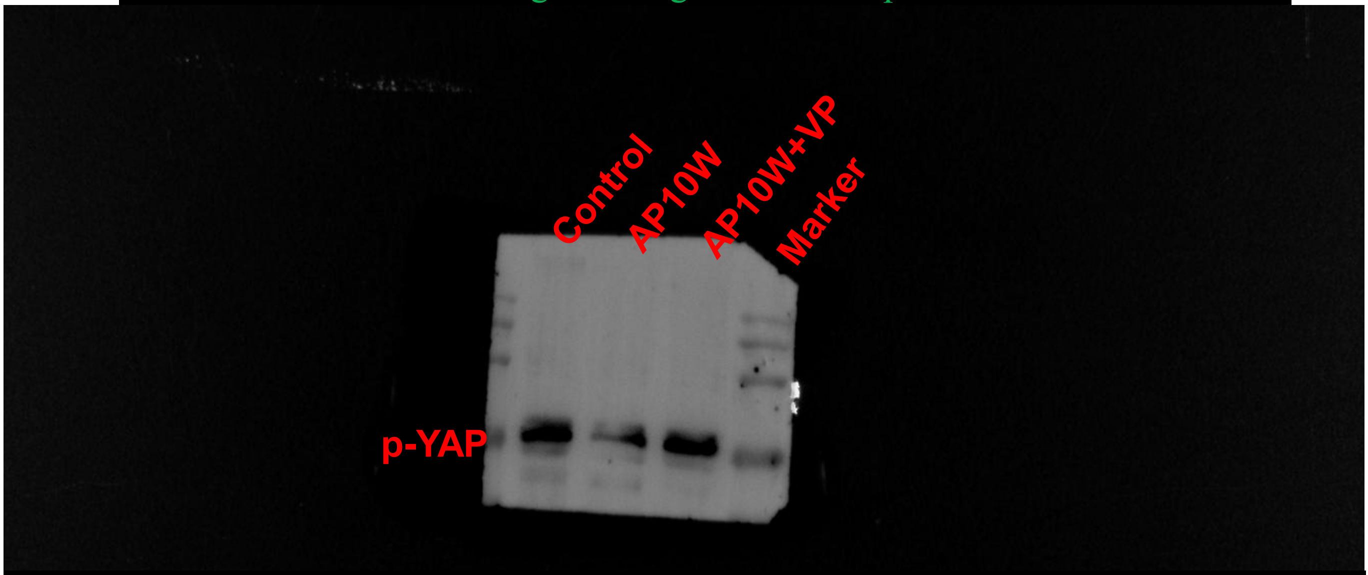

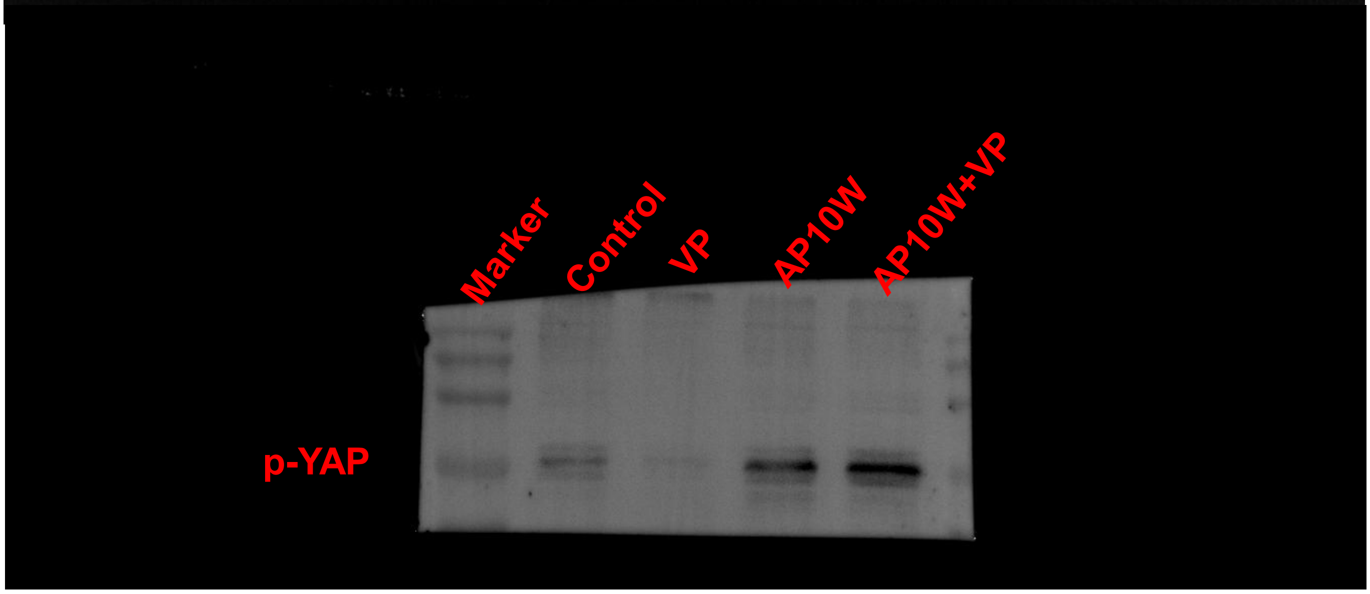

Supplement: Supplementary file 1 [file biomolecules-16-00720-s001.zip › Original Images for Blots.pdf]
