# Supplementary material for: The Multifunctional Peptide AP10W Enhances Skin Wound Healing Through Macrophage Reprogramming and Angiogenesis
Source: Biomolecules. 2026 May 13;16(5):720. doi: 10.3390/biom16050720 (PMC13204542; doi:10.3390/biom16050720)
Supplement: Supplementary file 1 [file biomolecules-16-00720-s001.zip › Table S1 comparing AP10W with other previously published proteins.pdf]

Table S1 comparing AP10W with other previously published proteins

Peptide	Reported Functions	Reference
LL-37	Wound closure promotion;<br>antimicrobial activity;<br>anti-inflammatory activity;<br>promotion of cell migration/proliferation;<br>tissue repair/remodeling-related effect;<br>angiogenesis/collagen remodeling/anti-biofilm/antioxidant activity.	[1-8]
Histatin-1	Promotion of epithelial cell migration;<br>wound closure/reepithelialization promotion;<br>pro-angiogenic activity reported in oral wound-healing context;<br>antimicrobial/antifungal activity mainly described for histatin family members, especially histatin-5;	[9-12]
Thymosin β4 / Tβ4	Promotion of keratinocyte/endothelial cell migration;<br>angiogenesis promotion;<br>anti-inflammatory activity;<br>wound closure/reepithelialization acceleration;<br>collagen deposition/remodeling and tissue repair modulation.	[13]
IDR-1018	Immunomodulatory activity; anti-inflammatory activity; broad-spectrum anti-biofilm activity; antimicrobial activity, usually modest/direct or context-dependent; potential benefit for infected wounds through host defense regulation and biofilm inhibition; direct promotion of keratinocyte/fibroblast migration, angiogenesis, collagen remodeling, or cutaneous wound closure.	[14-16]

Pexiganan / MSI-78	Broad-spectrum antimicrobial activity; clinical application for infected diabetic foot ulcers/wound infection control; wound-healing benefit mainly through bacterial burden reduction rather than direct pro-migratory/pro-angiogenic activity.	[17,18]
Human β-defensin-3 / hBD-3	Antimicrobial activity; immunomodulatory/chemotactic activity; promotion of keratinocyte migration/proliferation and epithelial repair-related responses; anti-inflammatory activity.	[19,20]
DRGN-1	Broad-spectrum antimicrobial activity; anti-biofilm activity, including activity against mixed-species biofilms; promotion of keratinocyte migration; acceleration of wound closure, including biofilm-associated wound models.	[21]
Esculentin-1a-derived peptide	Antimicrobial activity, especially against Pseudomonas aeruginosa; anti-biofilm activity; promotion of keratinocyte migration; wound closure promotion in in vitro wound models; anti-inflammatory/immunomodulatory effect.	[22,23]
Tiger17	Promotion of keratinocyte/fibroblast migration; promotion of cell proliferation; wound closure acceleration in skin wound models; anti-inflammatory activity.	[24]
Epinecidin-1	Broad-spectrum antimicrobial activity; activity against wound-infecting bacteria and infected wound protection; anti-inflammatory/immunomodulatory activity; promotion of infected wound healing mainly through infection control and inflammation modulation.	[25]
AP10W	Antimicrobial activity against both bacteria and fungi;	[26-27];

	anti-biofilm activity; anti-inflammatory/immunomodulatory activity; promotion of keratinocyte migration/proliferation and epithelial repair-related responses; promotes angiogenesis.	this study

Reference:

[1] Wnorowska, U.; Fiedoruk, K.; Piktel, E.; Prasad, S.V.; Sulik, M.; Janion, M.; Daniluk, T.; Savage, P.B.; Bucki, R. Nanoan-tibiotics containing membrane-active human cathelicidin LL-37 or synthetic ceragenins attached to the surface of magnetic nanoparticles as novel and innovative therapeutic tools: current status and potential future applications. J. Nanobiotech-nology 2020, 18, 3.

[2] Kahlenberg, J.M.; Kaplan, M.J. Little peptide, big effects: the role of LL-37 in inflammation and autoimmune disease. J. Immunol. 2013, 191, 4895-4901.

[3] Pletzer, D.; Hancock, R.E.; Antibiofilm Peptides: Potential as Broad-Spectrum Agents. J. Bacteriol. 2016, 198, 2572-2578.

[4] Agier, J.; Efenberger, M.; Brzezińska-Błaszczyk, E. Cathelicidin impact on inflammatory cells. Cent. Eur. J. Immunol. 2015, 40, 225-235

[5] Heilborn, J.D.; Nilsson, M.F.; Kratz, G.; Weber, G.; Sørensen, O.; Borregaard N, Ståhle-Bäckdahl M. The cathelicidin an-ti-microbial peptide LL-37 is involved in re-epithelialization of human skin wounds and is lacking in chronic ulcer epithe-lium. J. Invest. Dermatol. 2003, 120, 379-389.

[6] Steinstraesser, L.; Kraneburg, U.; Jacobsen, F.; Al-Benna, S. Host defense peptides and their antimicrobi-al-immunomodulatory duality. Immunobiology 2011, 216, 322-333.

[7] Koczulla, R.; von, Degenfeld, G.; Kupatt, C.; Krötz, F.; Zahler, S.; Gloe, T.; Issbrücker, K.; Unterberger, P.; Zaiou, M.; Lebherz, C.; Karl, A.; Raake, P.; Pfosser, A.; Boekstegers, P.; Welsch, U.; Hiemstra, P.S.; Vogelmeier, C.; Gallo, R.L.; Clauss M, Bals R. An angiogenic role for the human peptide antibiotic LL-37/hCAP-18. J. Clin. Invest. 2003, 111, 1665-1672.

[8] Gao, H.; Tang, F., Chen, B.; Li, X. LL-37 Attenuates Sepsis-Induced Lung Injury

by Alleviating Inflammatory Response and Epithelial Cell Oxidative Injury via ZBP1-Mediated Autophagy. Toxins (Basel). 2025, 17, 306.

[9] Oudhoff, M.J.; Bolscher, J.G.; Nazmi, K.; Kalay, H.; van 't Hof W; Amerongen, A.V.; Veerman, E.C. Histatins are the major wound-closure stimulating factors in human saliva as identified in a cell culture assay. FASEB J. 2008, 22, 3805-3812.

[10] Oudhoff, M. J.; Kroeze, K. L.; Nazmi, K.; van den Keijbus, P. A.; van 't Hof, W.; Fernandez-Borja, M.; Hordijk, P. L.; Gibbs, S.; Bolscher, J. G.; & Veerman, E. C. Structure-activity analysis of histatin, a potent wound healing peptide from human saliva: cyclization of histatin potentiates molar activity 1,000-fold. FASEB J. 2009, 23, 3928-3935.

[11] Torres, P.; Díaz, J.; Arce, M.; Silva, P.; Mendoza, P.; Lois, P.; Molina-Berríos, A.; Owen, G.I.; Palma, V.; Torres, V.A. The salivary peptide histatin-1 promotes endothelial cell adhesion, migration, and angiogenesis. FASEB J. 2017, 31, 4946-4958.

[12] Puri, S.; Edgerton, M.; How does it kill? understanding the candidacidal mechanism of salivary histatin 5. Eukaryot. Cell. 2014, 13, 958-964.

[13] Goldstein, A.L.; Hannappel, E.; Sosne, G.; Kleinman, H.K. Thymosin β4: a multi-functional regenerative peptide. Basic properties and clinical applications. Expert. Opin. Biol. Ther. 2012, 12, 37-51.

[14] Scott, M.G.; Dullaghan, E.; Mookherjee, N.; Glavas, N., Waldbrook, M.; Thompson, A.; Wang, A.; Lee, K.; Doria, S.; Hamill, P.; Yu, J.J.; Li, Y.; Donini, O.; Guarna, M.M.; Finlay, B.B.; North, J.R.; Hancock, R.E. An anti-infective peptide that selectively modulates the innate immune response. Nat. Biotechnol. 2007, 25, 465-472.

[15] Alencar-Silva, T.; Zonari, A.; Foyt, D.; Gang, M.; Pogue, R.; Saldanha-Araujo, F.; Dias, S.C.; Franco, O.L.; Carvalho, J.L. IDR-1018 induces cell proliferation, migration, and reparative gene expression in 2D culture and 3D human skin equiva-lents. J. Tissue Eng. Regen. Med. 2019,13, 2018-2030.

[16] de la Fuente-Núñez, C.; Reffuveille, F.; Haney, E.F.; Straus, S.K.; Hancock, R.E. Broad-spectrum anti-biofilm peptide that targets a cellular stress response. PLoS

Pathog. 2014, 10, e1004152.

[17] Lipsky, B.A.; Holroyd, K.J.; Zasloff, M. Topical versus systemic antimicrobial therapy for treating mildly infected diabetic foot ulcers: a randomized, controlled, double-blinded, multicenter trial of pexiganan cream. Clin. Infect. Dis. 2008, 47, 1537-1545.

[18] Ge, Y.; MacDonald, D.L.; Holroyd, K.J.; Thornsberry, C.; Wexler, H.; Zasloff, M. In vitro antibacterial properties of pexiganan, an analog of magainin. Antimicrob. Agents Chemother. 1999, 43, 782-788.

[19] Semple, F.; Webb, S.; Li, H.N.; Patel, H.B.; Perretti, M.; Jackson, I.J.; Gray, M.; Davidson, D.J.; Dorin, J.R. Human be-ta-defensin 3 has immunosuppressive activity in vitro and in vivo. Eur. J. Immunol. 2010, 40, 1073-1078.

[20] Niyonsaba, F.; Ushio, H.; Nakano, N.; Ng, W.; Sayama, K.; Hashimoto, K.; Nagaoka, I.; Okumura, K.; Ogawa, H. Anti-microbial peptides human beta-defensins stimulate epidermal keratinocyte migration, proliferation and production of proinflammatory cytokines and chemokines. J. Invest. Dermatol. 2007, 127, 594-604.

[21] Chung, E.M.C.; Dean, S.N.; Propst, C.N.; Bishop, B.M.; van Hoek, M.L. Komodo dragon-inspired synthetic peptide DRGN-1 promotes wound-healing of a mixed-biofilm infected wound. NPJ Biofilms Microbiomes. 2017, 11, 9.

[22] Di Grazia, A.; Cappiello, F.; Cohen, H.; Casciaro, B.; Luca, V.; Pini, A.; Di, Y.P.; Shai, Y.; Mangoni, M.L. D-Amino acids incorporation in the frog skin-derived peptide esculentin-1a (1-21) NH2 is beneficial for its multiple functions. Amino Acids. 2015, 47, 2505-2519.

[23] Di Grazia, A.; Cappiello, F.; Imanishi, A.; Mastrofrancesco, A.; Picardo, M.; Paus, R.; Mangoni, M.L. The Frog Skin-Derived Antimicrobial Peptide Esculentin-1a (1-21) NH2 Promotes the Migration of Human HaCaT Keratinocytes in an EGF Re-ceptor-Dependent Manner: A Novel Promoter of Human Skin Wound Healing? PLoS One 2015, 10, e0128663.

[24] Tang, J.; Liu, H.; Gao, C.; Mu, L; Yang, S.; Rong, M.; Zhang, Z.; Liu, J.; Ding, Q.; Lai, R. A small peptide with potential ability to promote wound healing. PLoS One.

2014, 9, e92082

[25] Huang, H.N.; Rajanbabu, V.; Pan, CY.; Chan, YL.; Wu, CJ.; Chen, JY.; Use of the antimicrobial peptide Epinecidin-1 to protect against MRSA infection in mice with skin injuries. Biomaterials. 2013, 34, 10319-10327.

[26] Gong, Y.; Li, H.; Wu, F.; Li, Y.; Zhang, S. Fungicidal Activity of AP10W, a Short Peptide Derived from AP-2 Complex Subunit mu-A, In Vitro and In Vivo. Biomolecules 2022, 12, 965.

[27] Gong, Y.; Li, H.; Wu, F.; Zhang, X.; Zhou, Y.; Zhang, S. A short peptide derived from zebrafish AP-2 complex subunit mu-A AP2M1A354–382 has antimicrobial activity against multi-drug resistant bacteria. Pept. Sci. 2021, 114, e24258.
