# Supplementary figures and images for: The Multifunctional Peptide AP10W Enhances Skin Wound Healing Through Macrophage Reprogramming and Angiogenesis
Source: Biomolecules. 2026 May 13;16(5):720. doi: 10.3390/biom16050720 (PMC13204542; doi:10.3390/biom16050720)

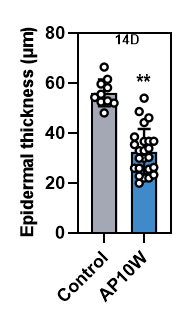

Supplement: Supplementary file 1 [file biomolecules-16-00720-s001.zip › Figure S1.tif]
